# A new terrestrial palaeoenvironmental record from the Bering Land Bridge and context for human dispersal

**DOI:** 10.1098/rsos.180145

**Published:** 2018-06-20

**Authors:** Matthew J. Wooller, Émilie Saulnier-Talbot, Ben A. Potter, Soumaya Belmecheri, Nancy Bigelow, Kyungcheol Choy, Les C. Cwynar, Kimberley Davies, Russell W. Graham, Joshua Kurek, Peter Langdon, Andrew Medeiros, Ruth Rawcliffe, Yue Wang, John W. Williams

**Affiliations:** 1 Water and Environmental Research Center, Institute of Northern Engineering, Fairbanks, AK, USA; 2 Alaska Stable Isotope Facility, College of Fisheries and Ocean Sciences, Fairbanks, AK, USA; 3 Department of Anthropology, Fairbanks, AK, USA; 4 Alaska Quaternary Center, University of Alaska Fairbanks, Fairbanks, AK, USA; 5 Laboratory of Tree Ring Research, University of Arizona, Tucson, AZ, USA; 6 Department of Biology, University of New Brunswick, Fredericton, New Brunswick, Canada; 7 Department of Geography and Environment, University of Southampton, Southampton, Hampshire, UK; 8 School of Geography, Earth and Environmental Sciences, Plymouth University, Plymouth, UK; 9 Department of Geosciences and Earth and Mineral Sciences Museum & Art Gallery, The Pennsylvania State University, University Park, PA, USA; 10 Department of Geography and Environment, Mount Allison University, Sackville, New Brunswick, Canada; 11 York University, Toronto, Canada; 12 Department of Geography, University of Wisconsin-Madison, Madison, WI, USA; 13 Center for Climatic Research, University of Wisconsin-Madison, Madison, WI, USA

**Keywords:** Beringia, stable isotopes, diatoms, cladocerans, chironomids, environmental change

## Abstract

Palaeoenvironmental records from the now-submerged Bering Land Bridge (BLB) covering the Last Glacial Maximum (LGM) to the present are needed to document changing environments and connections with the dispersal of humans into North America. Moreover, terrestrially based records of environmental changes are needed in close proximity to the re-establishment of circulation between Pacific and Atlantic Oceans following the end of the last glaciation to test palaeo-climate models for the high latitudes. We present the first terrestrial temperature and hydrologic reconstructions from the LGM to the present from the BLB's south-central margin. We find that the timing of the earliest unequivocal human dispersals into Alaska, based on archaeological evidence, corresponds with a shift to warmer/wetter conditions on the BLB between 14 700 and 13 500 years ago associated with the early Bølling/Allerød interstadial (BA). These environmental changes could have provided the impetus for eastward human dispersal at that time, from Western or central Beringia after a protracted human population standstill. Our data indicate substantial climate-induced environmental changes on the BLB since the LGM, which would potentially have had significant influences on megafaunal and human biogeography in the region.

## 1. Introduction

**Figure 1.**
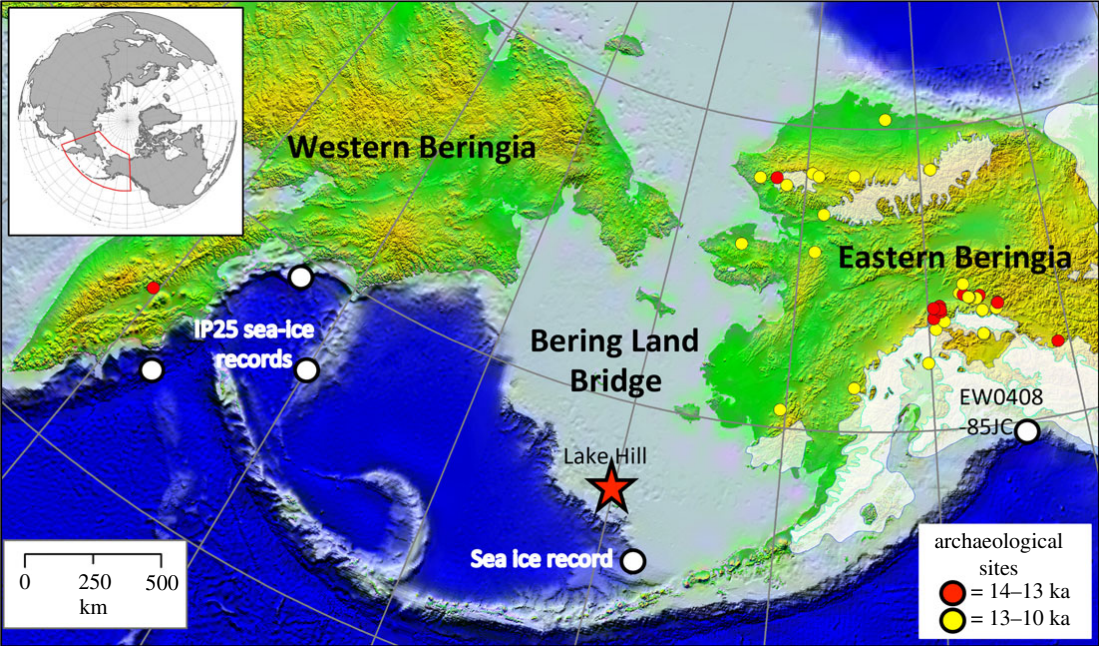
Locations of St. Paul Island, Alaska and other datasets referred to in the text [4–7].

Major dispersal events throughout human prehistory have been linked to sea level and other major environmental changes, such as changes in vegetation and moisture [1–3]. Beringia, a region that includes easternmost Asia, westernmost northern North America, and the now-submerged Bering Land Bridge (BLB) (figure 1), provided the setting for the exchange of plants and animals between the two continents throughout the Pleistocene as a result of repeated exposure of the BLB, and was the key entry point for human dispersal of Native American ancestors into the Americas [8–10].

The initial peopling of the Americas remains a topic of intense debate, with proposed evidence for first inhabitants widespread in time and space (e.g. [11–15]). However, new genetic and archaeological evidence indicate migration of Native American ancestors through Beringia sometime after 16 000 years ago, but the exact timing and specific routes remain unresolved [10,16]. Ancient Beringians, a newly discovered population of Native Americans, radiated from other groups approximately 20 000 years ago and are connected directly with the Denali complex, located throughout Alaska and adjacent regions between 12 500 and 6000 years ago. This population may represent a second migration event after the initial dispersal of humans through Beringia [10].

The modelled palaeoenvironmental context of human adaptation and dispersal across Beringia includes the important roles of temperature, moisture and vegetation [3], but remains speculative due to a distinct lack of empirical palaeoenvironmental data from the now-submerged BLB (figure 1). Most of the multiproxy data from Eastern Beringia covers the Holocene [17] rather than the Late Glacial [18]. While temperature and hydrological records from the now-flooded central BLB are non-existent due to flooding with sea-level rise (figure 1), a few vegetation records that date to the Last Glacial Maximum (LGM) or Late Glacial have been published, either from emergent islands (St. Paul and St. Lawrence [19–23]) or from marine sediment archives that reached BLB terrestrial sediments [8,24]. However, dating and stratigraphic uncertainties make some of these records difficult to interpret [19–21]. As a result, most inferences about past environmental conditions have come from Bering Sea marine records [4–6], climate and vegetation models [25], as well as lake and terrestrial sediments from the BLB's perimeter [26–32]. Most of these localities (with the exception of one [4], which is approx. 250 km away) are located at least 700 km distant, often more than 1000 km from our study site on the south-central BLB. Clearly, enhanced palaeoenvironmental information is needed from the BLB from the LGM to better define the physical setting allowing for an improved understanding of regional post-glacial biogeography, including early human movements through Beringia. Our study presents the first terrestrially-based reconstructions of temperature and hydrologic changes at a site that is firmly within the boundary of the now-submerged south-central BLB and that has remained exposed above sea level from at least the LGM [23] (figure 1). Moreover, our record is the first well-dated site located on the southern BLB itself that sheds light on climate change from approximately 18 000 years ago, a time period and locality that is critical for evaluating the ecological context for human dispersals into Alaska [10].

## 2. Material and methods

We analysed a suite of abiotic and biotic proxies of past environmental conditions (electronic supplementary material, S1) preserved in a dated [22,23] core of lake sediments taken from Lake Hill on St. Paul Island (figure 1). St. Paul Island is characterized by low relief, several freshwater lakes, no lotic systems and moderately productive moss-herbaceous tundra vegetation [22]. The chronology for the core was established using radiocarbon dating, and the vegetation history at the site was determined using pollen data, sedimentary ancient DNA and plant macrofossils [22,23]. We used a chironomid-based inference model [33] (electronic supplementary material, S1, S6 and S7) to reconstruct July air temperatures [31]. Diatom and cladoceran assemblage composition (electronic supplementary material, S8–S10) and oxygen isotopic analysis of chitinous chironomid headcapsules were used to infer hydrologic changes [22] (electronic supplementary material, S1). Samples for all palaeoecological and palaeoenvironmental proxies were taken at identical core depths, to minimize problems with proxy correlation. Extraction and processing of spores and pollen followed a modified version of the University of Minnesota LacCore protocol [34]. These modifications included the addition of 1 ml of polystyrene microspherule solution (5.0 × 10^4^ sph/ml ± 8%) to calculate pollen concentrations and accumulation rates. Each pollen sample was scanned at 400× magnification (or 1000× magnification with oil immersion, if necessary) and at least 300 pollen grains per sample were counted and identified. Pollen and spore abundances were expressed as accumulation rates (grains cm^−2^ yr^−1^).

Cladoceran, diatom and stable isotopic analyses were conducted at Alaska Stable Isotope Facility (ASIF), University of Alaska Fairbanks. Preparation of diatom samples followed a modified protocol [35] (see electronic supplementary material, S1) with each sample between 0.1 and 0.2 g of non-calcareous wet sediment treated with 5 ml of 30% hydrogen peroxide, which was left to digest for one week before being rinsed several times with distilled water. Dilutions of the resulting slurries were pipetted onto cover slips and left to dry at room temperature before being permanently mounted onto glass slides using Meltmount™, a thermal plastic with a refractive index similar to Naphrax (1.704). A minimum of 400 diatom valves were enumerated on random transects from each sample using a Leica DM microscope at 1000× magnification under oil immersion.

Cladocera were processed using previously published methods [36]. For cladocerans, sediment samples were deflocculated in a 10% KOH solution and the 125 and 65 µm fractions were isolated by sieving. The retained chitinous remains were transferred into vials using distilled water and stained with Safranin solution. Aliquots of 1 ml were pipetted onto a Sedgewick-Rafter cell and a minimum of 150 individuals for each sample were counted using a compound microscope at 400× magnification. Cladocerans were identified using keys to species (electronic supplementary material, S1), wherever possible. The most numerous sclerites (e.g. carapace, post-abdomen or head-shield) were employed to calculate the number of individuals of each species and expressed as percentage relative abundance.

Volumetric samples of lake sediment were processed for subfossil chironomid head capsules following standard procedures for non-calcareous sediments [31] (electronic supplementary material, S1). First, sediments were deflocculated by gently warming in a 5% KOH solution for approximately 30 min. Sediments were then rinsed with distilled water on a 95 µm mesh sieve. The remaining material retained on the sieve was then transferred into a beaker with approximately 25 ml of distilled water. Successive aliquots were next poured into a Bogorov counting tray and all head capsules were handpicked using fine forceps at approximately 25× magnification. To ensure all subfossil chironomids were collected, multiple passes (i.e. two focused on the surface and two focused on the bottom of the Bogorov tray) were made through each aliquot. Subfossils were then placed on a cover glass and permanently mounted on a slide using Entellan^®^. Chironomids were identified with brightfield illumination and a compound microscope at either 200× or 400× magnification. Identification of head capsules followed standard subfossil taxonomic keys for chironomid subfamilies (see electronic supplementary material, S1).

The oxygen isotope analyses of chironomid headcapsules were conducted using previously published methods [37]. After freeze-drying, samples were placed in an auto-sampler attached to a TCEA-IRMS system for analysis. All δ^18^O values are expressed relative to Vienna Standard Mean Ocean Water (VSMOW) and have an analytical precision of 0.1 per mil (‰). Bulk sediment samples were collected from the core at 16 cm intervals for isotopic and other geochemical analyses, and processed according to previously published protocols [38] (electronic supplementary material, S1).

## 3. Results

**Figure 2.**
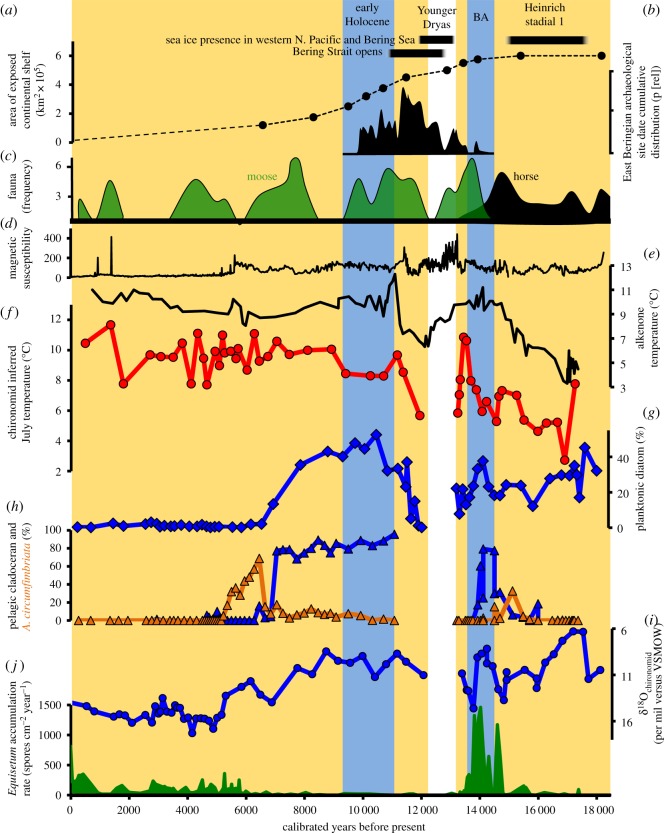
Palaeoenvironmental proxy data from St. Paul Island, Alaska and the vicinity referred to in the main text (sea ice presence = [5,6], Bering Strait opens = [8,39], (*a*) change in BLB surface area [40]; (*b*) archaeological dates for Alaska between 16 000 and 9000 years ago; (*c*) frequency of calibrated radiocarbon ages moose (+, break in frequency scale up to 17 at this point) and horse for interior Alaska (see electronic supplementary material,); (*d*) magnetic susceptibility of Lake Hill sediments; (*e*) alkenone temperature in the N. Pacific; (*f*–*j*) from Lake Hill, respectively = chironomid-inferred July temperature, planktonic diatoms % of assemblage, cladoceran data, oxygen isotope analyses of chironomid head capsules, *Equisetum* spore accumulation rates. No cladocerans or chironomids were evident during the YD in the St. Paul core and only trace amounts of freshwater diatoms (see main text for explanation). (Note the reversed scale for δ^18^O values.)

The palaeoenvironmental reconstruction from Lake Hill extends from 18 500 years ago to the present (figure 2) and provides, for the first time, multiproxy-based evidence from the BLB to compare with existing human biogeographic information. The Late Glacial portion of the Lake Hill record reveals a period of relatively warm and wet conditions, based on the chironomid temperature reconstruction, δ^18^O values, diatom, cladoceran and *Equisetum* spores, during the Bølling/Allerød (BA—approx. 14 600 to approx. 14 000 years ago) [41] that is bracketed by the cold and dry conditions associated with both Heinrich Stadial 1 (HS1; from 18 500 to approx. 14 600 years ago) and the Younger Dryas (YD; from 12 900 to approx. 11 650 years ago) [41–43] (figure 2). Existing vegetation records from the LGM at the BLB margins indicate a herbaceous vegetation, though a few willow and birch shrubs may have been present [26–29,31,32]. The climate was cold and dry, as indicated by the near absence of shrubs and the abundance of taxa prevalent on exposed settings (cf. *Kobresia* on the Seward Peninsula [28] as well as the paucity of lake sediments that date to this period (such as Burial lake, which was dry approx. 34 000–23 000 years ago [31,32]).

The extent to which the now-flooded BLB was relatively mesic at the LGM compared to its margins remains unclear. Elias *et al*. [8], analysing cores from just south of the Seward Peninsula, provided evidence for shrubs and ponds on the BLB; however, their dataset spanned approximately 24 000–17 000 years ago and some samples date to late in the period, when climate had begun to ameliorate. By contrast, a core from Norton Sound [24] indicated a herbaceous tundra with few shrubs during the LGM. The portion of the record we present from St. Paul Island between approximately 18 000 and 15 000 years ago indicates the site was progressively drying and relatively cold (figure 2). Chironomid-inferred July air temperatures from Lake Hill average about 5°C for this period, a minimum for the record that closely agrees with previously published alkenone temperature estimates [5] (figure 2). There is also evidence of seasonal sea-ice expansion in the Bering Sea and the western North Pacific during HS1 [6] (figure 2). By approximately 16 000 years ago, some sites at the BLB margins indicate expansion of birch shrubs [26,29], while others indicate a later birch expansion, about 14 000 years ago [32]. This difference is presumably an artefact of dating methods and the younger age is more accurate [44]. The younger age is also consistent with the termination of HS1. Increasing aridity on the south-central BLB during HS1 is supported by multiple indicators of declining lake levels at Lake Hill from approximately 17 700 until approximately 15 000 years ago, based on the diatom and cladoceran assemblages (see also electronic supplementary material, S1) (figure 2). This includes a trend towards higher δ^18^O values (figure 2), denoting increasing evaporative losses from Lake Hill (electronic supplementary material, S1 and S2) severe enough to promote a decline in relative abundance of freshwater, planktonic diatoms and pelagic cladocerans, and an increase of a high conductivity-tolerant aquatic invertebrate species (i.e. *Alona circumfimbriata*) [45] by the end of HS1 (figure 2).

From approximately 14 600 to 14 000 years ago, based on the diatoms, cladocerans, δ^18^O values, chironomids and *Equisetum* spores, we document a relatively abrupt and short-lived period of increased moisture and warmer conditions on the south-central BLB, which corresponds to the BA (figure 2). This evidence includes nearly synchronous increases in pelagic cladocerans and planktonic diatoms, along with decreasing δ^18^O values, resulting from diminished evaporative losses from Lake Hill. Planktonic diatom species present during the BA include *Cyclotella tripartita* and *Asterionella formosa*, which in mainland Alaska lakes are observed to have water depth optima between 9.4 and 15.6 m [46], far deeper than the approximately 1.5 m water depth of Lake Hill today (electronic supplementary material, S3). Although diatom assemblages can be influenced by changes in nutrients, these particular diatom species point to deeper water. These changes co-occur with heightened vegetation productivity inferred from increased total pollen accumulation rates [23] and most notably by a marked increase in *Equisetum* spores (figure 2), consistent with enhanced moisture in the area at this time. The BA also saw a marked rise in chironomid-inferred July temperatures from approximately 5°C to approximately 11°C (figure 2) on St. Paul Island and of sea surface temperature (SST) in the northern North Pacific [5,6], which accompanied a diminished extent of seasonal sea ice in the Bering Sea [4,6].

The relatively short-lived, warm and wet BA period on the south-central BLB was followed by a marked shift to dry and cold conditions, inferred from diatoms and chironomids, associated with the YD between 12 900 and 11 700 years ago (figure 2). This period of decreased temperatures over the Northern Hemisphere [47] was accompanied by an expansion of seasonal sea-ice extent into the Bering Sea [4] and western North Pacific [6], and decreasing July temperatures and SST in the northern North Pacific [5] (figure 2). Lake levels at St. Paul Island declined so substantially during the transition from the BA into the YD that layers of loess and sand accumulated in the Lake Hill basin, containing insufficient remains of aquatic organisms to allow environmental inferences (figure 2). Trace amounts of well-preserved freshwater diatoms and no evidence of palaeosols in the Lake Hill record during the YD indicate that an ephemeral aquatic habitat may have existed but was regularly inundated with loess and sand. These YD-dated sediments also contained the degraded remains of marine diatoms (electronic supplementary material, S4), likely resulting from aeolian redeposition into the Lake Hill crater from deflating marine sediments from the dry, cold and exposed central BLB at this time. Degradation of these marine diatom frustules likely resulted from the same high wind velocities noted as scouring ventifacts found in archaeological sites in Alaska dating to the YD period [7]. These YD-dated sediments also have very low organic content and carbon to nitrogen ratios (C : N values) (electronic supplementary material, S5), implying low terrestrial productivity (e.g. [48]). The terrestrial pollen assemblages have very low pollen accumulation rates, and high abundances of non-local pollen types (*Picea*, *Pinus*), also indicating extremely low pollen and vegetation productivity on St. Paul Island during the YD [23]. The July temperature reconstructions both into and out of the YD indicate that cold summer conditions prevailed (figure 2).

The onset of the Holocene at Lake Hill documents an increase in chironomid-inferred July temperatures, and high moisture availability reflected by a rapid and substantial increase in planktonic diatoms and pelagic cladocerans, as well as diminished evaporative losses inferred from decreasing δ^18^O values (figure 2). The planktonic taxa include those that are favoured at levels deeper than present lakes (electronic supplementary material, S1). With the exception of the temperature reconstruction presented here, a detailed interpretation of the palaeoenvironmental reconstruction for the Holocene section was presented by Graham *et al*. [22] and Wang *et al*. [23], which drew attention to a marked decrease in lake levels after approximately 8000 years based on the diatom, cladoceran and oxygen isotope data. Lake levels decreased so substantially by approximately 6000 years so as to cause a local extinction of St. Paul Island mammoths due to limited access to freshwater resources [22]. The temperature reconstruction for the Holocene on St. Paul indicates a general increasing trend from approximately 8°C towards the start of the Holocene to a high of approximately 11.5°C in the last 1000 years. Although the temperature record does not indicate a marked temperature change during the mid-Holocene when lake levels were at their lowest, this period of lower lake levels does appear to correlate with a middle Holocene Thermal Maximum in Eastern Beringia [17].

## 4. Discussion

This new multiproxy palaeoenvironmental dataset allows a meaningful reconstruction of the Late Glacial environmental context across the BLB when humans were first dispersing into Alaska [10]. A wide range of genetic studies indicate a human population standstill between 25 000 and 16 000 years ago, during which Native American ancestors became genetically isolated from East Asians [16,49]. The location of this standstill is debated; some researchers have argued for interior northeastern Asia on the basis of archaeological evidence for human occupation during this period [7,50,51], while others have suggested Beringia based on human niche models [3]. Other studies point to the southern coast of central Beringia based on indirect estimates of biological productivity [7,9,16,49–52].

Analyses of mtDNA indicate population expansion of humans related to post-LGM colonization of the Americas sometime after 15 900 years ago, with 95% confidence intervals of 17 325–11 500 years ago [16]. The location of this ancestral population prior to this expansion remains unknown. However, it is likely to have been in Asia because there is evidence of human occupation there between 25 000 and 16 000 years ago, whereas there is still limited evidence of humans being present in the Americas during this period [7,50,51]. This migration period encompasses expansion north and east into Beringia, as well as further expansion south into North and South America. Since two lineages of Native Americans were present by 17 000 years ago (Ancient Beringians and North and South Native Americans) [10], this raises the possibility of multiple migrations. By 12 600 years ago, the Southern Native American lineage is anchored by the Anzick child in Montana, associated with the widespread Clovis complex. This suggests that Ancient Beringians, who apparently remained in Beringia through the early Holocene, were a later migration [10]. Although the location of Ancient Beringian populations prior to 11 500 years remains unresolved, they may have split from other Native Americans around 20 000 years ago in Asia (Scenario 1) or, less likely, Alaska (Scenario 2). Current archaeological and palaeoecological data support the former [10]. There is a clear archaeological signal of expansion of people in Northeast Asia using Diuktai-related technologies after 17 000 years ago, connected with the earliest unequivocal site in Eastern Beringia, Swan Point, dating to 14 200 years ago. While human occupations have been asserted for LGM times in North America [12], or pre-LGM, or even 130 000 years ago [13], these remain equivocal [14,53]. More support has been given to sites dated to approximately 14 600–13 300 years ago, including Monte Verde and Paisley Caves [11], though some have also been criticized [54–56]. While the technological connections between Diuktai culture and Palaeoindian complexes is unclear [57,58], there was an unequivocal expansion of early Palaeoindians using Clovis and related technologies between 13 400 and 12 680 years ago. These demographic patterns require explanation, and we evaluate here the relationships between the timing of human occupations in Alaska (from approximately 14 500 years ago) and potential palaeoenvironmental drivers or influences that could explain them.

The increasing aridity trend recorded on the south-central BLB between approximately 17 700 until approximately 15 000 years ago is much the same period, from 18 500 to 15 000 years ago, that encompasses the latter part of the proposed standstill of ancestral Native American populations based on ancient DNA data [10,16]. Furthermore, the archaeological record indicates regional depopulation of Northern Siberia at this time [59]. The Lake Hill climatic inferences are also consistent with evidence for the relatively cold, dry and windier conditions on mainland Alaska at this time [60–63] and a period when Beringian horse (*Equus lambei*) is evident in interior Alaska (figure 2), taking advantage of an expansion in dryland, grazing habitats at this time [64]. Although archaeological sites in Siberia persist south of 58°N [65], evidence indicates that humans had not been migrating into the BLB region at the time of sustained cold and increasingly dry environmental conditions during the LGM and early deglaciation.

The Lake Hill data supports the hypothesis that expansion of humans into Alaska during the BA was associated with the widespread development of warmer and wetter conditions on the south-central BLB (figure 2), following similar trajectories as described for the end of the YD, approximately 11 650 years ago [66]. This environmental setting allowed humans associated with the earliest unequivocal occupations in Alaska (approx. 14 200–13 100 years ago) to exploit a wide range of resources, including wetland species (waterfowl and fish) and possibly salmon (with direct evidence of exploitation after 11 800 years ago), as well as large ungulates (bison and wapiti) [58,63,67]. Warmer and wetter conditions associated with the BA and early Holocene may also have affected the timing of eastward and westward dispersal of some animal species, such as moose (*Alces alces*), from the BLB [66,68]. Moose, notably, appear in interior Alaska during this widespread wet and warm period on the BLB associated with the BA (figure 2). Their range expanded from Asia during warmer and wetter intervals [69], which ultimately took them into Alaska during the warm and wet BA. In contrast the dryland, grazing species, such as horse (figure 2), mammoth and bison, diminished in abundance in Eastern Beringia at that time [64,68].

Human dispersal across the BLB is posited to have been both a push and pull phenomenon [63]. The ultimate ‘push’ factor was the rising sea level that accompanied the release of water from melting ice sheets leading to the eventual and complete flooding of the BLB, which accelerated at the beginning of the Holocene, around 11 500 years ago [63]. However, a chief environmental ‘pull’ for early eastward human and megafaunal dispersal across Beringia may have been ameliorating climate after the LGM, most notably the seasonal changes in moisture [3] associated with deglaciation that created suitable habitats for Northeast Asian hunter–gatherers [63], along with other environmental changes on the BLB and in Eastern Beringia [7,31,68].

The warm and wet conditions on the BLB during the BA may also have promoted the development of some less favourable conditions for humans and the resources they relied upon on the BLB, which could have provided some impetus to move out of central Beringia. It has been postulated that even during the cold and dry LGM, the relatively low-lying BLB acted as a mesic barrier, inhibiting the dispersal of some open-habitat, faunal species between Eastern and Western Beringia [68,70]. Marked changes in moisture have been identified as an important attribute to megafaunal turnover in Beringia [22,71] and human niche dynamics in the Palaeoarctic [3]. The mesic barrier proposed for the BLB during the LGM would have greatly expanded into much wider-spread wetlands and peatlands with the progressively warmer and wetter conditions during the BA, evident at the site of St. Paul Island. This would have reduced suitable habitats for large grazers (bison, horse, mammoth) [64] and would have either promoted their dispersal into other more favourable habitats in Beringia, such as uplands, or resulted in population declines, such as those seen for horse in interior Alaska (figure 2), due to the reduction and fragmentation in available grass-dominated habitats [64,71]. Ultimately, the widespread increase in moisture after the LGM contributed to the extinction of some of these grazing mega-herbivores [64,71]. These moist environments would also have hindered human ability to travel across the relatively low-lying landscape on the BLB and prompted them to seek easier and better-drained terrains in interior Alaska, such as the channels of major rivers [7,58]. The *Equisetum* spike in the Lake Hill record (figure 2) is also consistent with an increased prevalence of wetland vegetation, at least locally. Additional environmental factors ‘pushing’ humans and some faunal taxa out of the relatively low-lying BLB region during the BA may have included an increased proliferation of mosquito populations (both in abundance and in the longer duration of the season), which are abetted by warmer and wetter conditions [72]. Modern caribou herd movements are strongly influenced by the intensity of mosquito populations [73,74] and caribou populations can be inhibited by the development of wetter and warmer conditions [75,76]. Movement of caribou and other faunal resources that were available to humans on the BLB could have subsequently prompted humans to move from the BLB as they tracked the geographical shift in the availability of these resources.

The greatest rate of land area loss from the BLB due to sea-level rise after the LGM occurred between approximately 10 000 and 8500 years ago [40] (figure 2), which is at least 4500 years later than the prevalent archaeological evidence for the earliest human presence in Alaska [16]. This substantial temporal lag, combined with the well-constrained timing of environmental changes on the BLB preserved in the St. Paul Island record, suggests that sea-level changes did not provide an initial environmental ‘push’ corresponding to the earliest archaeological evidence of human presence in Alaska. With the opening of the Bering Strait due to sea-level rise [6,39], came a notable amelioration in environmental conditions on St. Paul Island, the Bering Sea [4] and the northern North Pacific (figure 2) due to oceanographic changes and a maximum in Northern Hemisphere summer insolation [25,77,78]. Conditions reverted to warm and humid in Eastern Beringia during the early Holocene, which followed the cold and dry YD. At Lake Hill, July air temperatures and relative moisture availability increased at this time (electronic supplementary material, S1). These changes on St. Paul Island (which became isolated from the mainland around 13 000 years ago) are accompanied by a similarly rapid increase in SST in the northern North Pacific [5] and a decrease in the extent of seasonal sea ice in the Bering Sea and western North Pacific [6] (figure 2). Our evidence for warm early Holocene July air temperatures for the south-central BLB is also consistent with existing palaeoecological evidence for relatively warm Late Glacial summers on the BLB, compared with modern (onshore) values [31,79], and corresponding to the time of maximum summer insolation at 60° N [80,81]. Archaeological data from interior Alaska currently indicate a decline in occupation sites during the YD, particularly between 12 900 and 12 500 years ago (only one known site), followed by a relatively large increase in occupations after the YD, at the beginning of the Holocene (14 known sites between 11 500 and 11 000 years ago) [57], coupled with a diversification in resources used by humans in Eastern Beringia [66]. This increase in archaeological sites also correlates with the emergence of a new cultural group: the Denali complex or Palaeoarctic tradition, that is genetically linked with Ancient Beringians, and may represent their initial migration into Alaska [10]. Rapid climatic changes following the YD have previously been related to human dispersal into sites above the Arctic Circle in Alaska, termed Northern Palaeoindians [40], and to faunal turnover in Beringia [66].

## 5. Conclusion

Palaeoenvironmental factors have been postulated to have had marked consequences for human dispersal into the Americas [3] and for faunal biogeography [22,66,71]. Our new multiproxy-based temperature and hydrologic reconstructions from a terrestrial site located on the south-central BLB revealed relatively warm and wet conditions during the BA, which validate this theory. We argue that these conditions could have provided environmental impetus for human movement, both across Beringia and, potentially, out of low-lying regions in the south-central BLB. The earliest archaeological evidence for the presence of humans in Alaska (approx. 14 200 years ago) thus corresponds with ecosystem, temperature and hydrologic changes in central and Eastern Beringia, and faunal range expansion into Eastern Beringia [66] during the BA, rather than with rising sea levels and the flooding of the BLB in later times. Some have argued for an inverse relationship between warmer climate and presence of human populations between 12 500 and 7000 years ago in Eastern Beringia [76]. The Lake Hill data supports this idea for some ‘push’ impetus for human dispersal out of the south-central BLB and into interior Alaska during a warmer period. Human movement out of the BLB could have been related to paludification and the development of extensive wetlands in the relatively low-lying terrain of this region, which might have impeded mobility and perhaps promoted intensified harassment of humans and mega-fauna by mosquitoes during the warm and wet BA. Although cultural mechanisms driving adaptations to climate change no doubt also have significance in the timing of post-glacial human migration into Alaska, this type of information remains elusive. Therefore, our results underscore the need for a deeper understanding of past environmental variability and timing in Beringia, to shed more light on the region's complexity of early human archaeology.

## Supplementary Material

Supplementary methods and results

## Data accessibility

Our data are deposited at the National Science Foundations' Arctic Data Center and have been uploaded at: https://arcticdata.io/catalog/#view/doi:10.18739/A27N5S.
