# Supplementary material for: A new terrestrial palaeoenvironmental record from the Bering Land Bridge and context for human dispersal
Source: R Soc Open Sci. 2018 Jun 20;5(6):180145. doi: 10.1098/rsos.180145 (PMC6030284; doi:10.1098/rsos.180145)
Supplement: Supplementary methods and results [file rsos180145supp1.docx]

**SI 1: SUPPLEMENTARY TEXT**

**Study site description and oxygen and hydrogen isotope water analyses:**

**Methods:** This study presents the first terrestrially-based reconstructions of temperature and hydrologic changes since the LGM from St. Paul Island (SI 2) in the Bering Sea, a site that was near the southern coastal margin of the now submerged BLB and has remained exposed above sea level since the LGM. St. Paul Island is characterized by low relief (max elevation 203 m above sea level), a few freshwater lakes, no springs or streams, and moderately productive moss-herbaceous tundra vegetation (1). We analyzed a suite of proxies of past environmental conditions preserved in a dated (1, 2) core of lake sediments taken from Lake Hill on St. Paul Island (Fig.1). The chronology for the core was previously established using radiocarbon dating and tephrochronology (1, 2) and the vegetation history at Lake Hill was previously determined using pollen data, ancient DNA and plant macrofossils (1). Mean precipitation for AD 2005 to 2014 was 540 mm per year (+68 mm) (3).

Water samples were collected from lakes on St. Paul Island (SI 2) and from precipitation events that occurred during field work in both March 2013 and June 2013. All water samples were transferred to glass vials and capped with no headspace for transport to the Alaska Stable Isotope Facility at the University of Alaska Fairbanks. A thermal conversion elemental analyzer (TC/EA) attached via a Conflo III to a Thermo Delta V Plus isotope ratio mass spectrometer (IRMS) was used to generate δD and δ^18^O values of water samples in accordance with previously published protocols (4). Analytical precision (expressed as one standard deviation from the mean) was 2‰ and 0.2‰ for δD and δ^18^O values, respectively. All δD and δ^18^O values for water are reported in per mil (‰) relative to Vienna Standard Mean Ocean Water (VSMOW). Lake water characteristics at Lake Hill were measured in March (beneath lake ice), including salinity, dissolved oxygen and pH using a YSI water quality meter. Lake depth was measured at the central coring site using a weighted measuring tape. Modeled values for mean monthly δD and δ^18^O values at the Lake Hill site were generated using an online database (5).

**Results:** The summer and winter lake-water δ^18^O and δD values from Lake Hill plot below the global meteoric water line (GMWL), and are significantly higher than the mean modeled annual precipitation for the site (SI 2). This isotopic pattern is also evident from the summer lake-water samples taken from lakes in interior Alaska that experience significant loss of lake water through evaporation (6). The likelihood that Lake Hill’s isotopic characteristics could be driven by input from the relatively higher δ^18^O and δD values of marine waters (SI 2) is ruled out by Lake Hill’s very low salinity (SI 3). These findings, along with the lake’s relatively shallow depth (1.3 m) relative to its size and closed catchment geomorphology (i.e. within a volcanic crater rim), indicate that the Lake Hill’s δ^18^O and δD values are influenced by the degree of evaporation. The site’s relatively high-elevation makes a marine incursion very unlikely.

**Spores and pollen:**

**Methods:** Complete and detailed discussions of the pollen and spores (along with plant macrofossils and plant aDNA results) have been previously published (1). We present here a reanalysis of selected findings from this study. Initial sampling resolution for spore and pollen was at 16 cm intervals throughout the core but with higher resolution sampling for some selected intervals (for example 4 cm and 2 cm resolution at 480-493 cm and 493-520 cm, respectively, to detect the timing of woolly mammoth extinction (2)). In total, 81 1-cm^3^-sediment samples were selected for pollen and spore analysis. Extraction and processing of spores and pollen followed a modified version of the University of Minnesota Limnological Research Center protocol (1). These modifications included the addition of 1 mL of polystyrene microspherule solution (5.0×10^4^ sph/mL ± 8%) to calculate pollen concentrations and accumulation rates. Each pollen sample was scanned at 400 × magnification (or 1000 × magnification with oil immersion, if necessary) and at least 300 pollen grains per sample were counted and identified. Pollen and spore abundances were expressed as accumulation rates (grains yr^-1^ cm^-2^).

**Results:** The pollen and spore records at Lake Hill suggest a stable, forb-graminoid tundra with a minor prostrate shrub component on St. Paul Island during the last 18,000 years (1), indicating generally cold environment. Although *Picea, Betula* and *Alnus* pollen grains were found in the core, the low pollen accumulation rates (<100 grains yr^-1^ cm^-2^), sedimentary ancient DNA, and plant macrofossils all indicate that these woody taxa were not present on the island and the southern coastal Bering Land Bridge (1). Accumulation rates for *Equisetum* spores were less than 80 grains yr^-1^ cm^-2^ before 14,700 years ago, but jumped to nearly 200 grains yr^-1^ cm^-2^ in 14,700 to 13,500 years ago. The *Equisetum* peak indicates a moss tundra and wetter climate during the BA. Poaceae accumulation rates were higher during the late glacial period, while accumulation rates were higher for Arctic forbs during the Holocene. Poaceae is the most abundant pollen taxon (~425 grains yr^-1^ cm^-2^), *Salix* is the most abundant shrub pollen taxon (~316 grains yr^-1^ cm^-2^), and *Artemisia* is the most abundant forb taxon (~266 grains yr^-1^ cm^-2^) (1).

**Diatoms:**

**Methods:** Preparation of diatom samples followed a modified protocol (7) with each sample between 0.1 and 0.2 g of non-calcareous wet sediment treated with 5 mL of 30% hydrogen peroxide, which was left to digest for one week before being rinsed several times with distilled water. Dilutions of the resulting slurries were pipetted onto cover slips and left to dry at room temperature before being permanently mounted onto glass slides using Meltmount™, a thermal plastic with a refractive index similar to Naphrax (1.704). A minimum of 400 diatom valves were enumerated on random transects from each sample using a Leica DM microscope (Laboratoire de Paléoécologie Aquatique, Université Laval) at 1000 × magnification under oil immersion. Keys consisting mainly of northern North American floras (8-10) assisted in identification to the lowest possible taxonomic level.

**Results:** The sedimentary diatom assemblages (SI 4,8,9) reflect the successive environmental changes that occurred in and around Lake Hill over time. Shortly after the LGM, during HS1, Lake Hill was a deep lake, dominated by planktonic species including *Discostella pseudostelligera*, *Cyclotella tripartita* and *Asterionella formosa*. In mainland Alaskan lakes, these taxa were observed to have water depth optima between 9.4 and 15.6 m (11). They are also associated with oligotrophic to mesotrophic waters. Other components of the assemblage during HS1 point to the presence of macrophytes (indicated by the epiphyte *Achnanthidium minutissimum*) and of a wetland in the lake’s catchment (indicated by the presence of the geni *Pinnularia* and *Stauroneis*). Overall, this assemblage points to relatively humid conditions.

At the start and later during the BA, tychoplanktonic taxa are abundant in the assemblages, notably the *Staurosirella pinnata* and *Pseudostaurosira brevistriata* complexes. These taxa are known to thrive in unstable conditions. They can tolerate highly variable light, nutrients, conductivity and turbulence. This zone is punctuated (at 14,210 years ago) by a peak in the relative abundance of the planktonic taxon *Cyclotella bodanica* *sensu lato* (16%). The presence of this species is associated with deep summer mixing (12). Greater mixing depths in the summer can be caused by several environmental variables, including increased wind strength, decreased solar radiation and inputs of terrestrially-derived dissolved organic matter. This species is known to have an optimum mixing depth of about 14 m in North American lakes, but it has also been found that the occurrence of the highest concentrations of cells of this species are driven by a combination of a mixing depth around 9 m and high nitrate concentrations around 60 µg.L-1 (12). Therefore, it is possible that this very punctual appearance of *Cyclotella bodanica* *sensu lato* at a high density could be due to a pulse of nutrient in-wash from a terrestrial source, triggered perhaps by a large flash flood-style series of rain events during that period.

The early YD is marked by a decrease in planktonic and tychoplanktonic taxa. Then, freshwater diatoms disappear and are replaced by heavily broken-up frustules of marine diatoms (SI 4). We interpret this as corresponding to a very dry period of loess accumulation (see main text). During the latter part of the YD, freshwater diatoms reappear in a peculiar assemblage succession, first a short-lived assemblage of planktonic taxa associated with high nutrient levels (*Stephanodiscus minutulus* and *Aulacoseira subarctica*), suggesting a pulse of water and nutrients from the catchment into the lake basin. This is followed by an (also short-lived) assemblage of tychoplanktonic taxa (as above) and benthic taxa associated with high nutrients and conductivity (esp. *Achnanthes carissima*).

The passage to improved climatic conditions in the early Holocene is marked by the increase and strong dominance (30-40% of the assemblage) throughout this period of the small centric diatom *Discostella pseudostelligera*. This taxon has a depth optimum of 9.4 m, and intermediate conductivity and nutrient preferences (see (11), as *Cyclotella pseudostelligera*). Moreover, *Discostella stelligera*, which is taxonomically and ecologically similar to *D. pseudostelligera*, is favored by a strongly stratified water column in summer, supporting thermal increase during that period. The benthic taxon *Stauroforma exiguiformis* becomes abundant in the assemblage and remains a dominant species in the lake until today. It could indicate high transparency of the water column, or that there were well-developed shallow areas within the lake at that time. Around 7000 cal yr BP, at the end of the early Holocene, the diatom assemblage points to a sharp decrease in lake depth reflecting a period of very dry and possibly cooler climate (2).

**Cladocerans:**

**Methods:** Cladocera were processed using an adapted version of a previously described method (13). Sediment samples were deflocculated in a 10% solution of potassium hydroxide and the 125 and 65 μm fractions were isolated by sieving. The retained chitinous remains were transferred into vials using distilled water and stained with Safranin solution. Aliquots of 1 mL were pipetted onto a Sedgewick-Rafter cell and a minimum of 150 individuals for each sample were counted using a compound microscope at 400 × magnification. Cladocerans were identified using keys (14-17) to species, wherever possible. The most numerous sclerites (i.e. carapace, post-abdomen or head-shield) were employed to calculate the number of individuals of each species and expressed as percentage relative abundance. This separation of the exoskeletal remains can make it difficult to identify certain taxa to species level. For the purposes of this study, *Bosmina longirostris* (Müller 1785) and *Eubosmina longispina* (Leydig 1860) were grouped as *Bosmina* spp., as the species can only be distinguished from the positioning of the lateral head pore, which is not always visible. Unidentifiable small and medium-sized *Alona*-type head shields and carapaces were grouped as *Alona* spp., as were *Chydorus* spp., and *Daphnia pulex* agg.

**Results:** The earliest cladoceran assemblages after the LGM indicate that a permanent waterbody existed on Lake Hill, dominated by *Chydorus* spp. (>76%), a littoral taxa (13) with plastic ecological preferences that can extend to the pelagic in nutrient rich systems (18) (SI 10). Shallow lakes are typically smaller with a greater proportion of littoral to pelagic habitat than deeper lakes, though many cladoceran taxa can exhibit plasticity. Aquatic mosses, filamentous algae and *Pediastrum* were also prevalent, along with *Hydracarina* and Coleoptera fragments. Towards the end of HS1, a rise in the abundance of conductivity tolerant *A. circumfibriata* and pelagic *Daphnia pulex* agg., coupled with the persisting presence of several *Alona* spp., especially *A. quadrangularis* and *A. guttata*, and an increase in the obligate benthic *Camptocercus* sp., suggests fluctuating open water lake levels with the presence of shallower littoral habitat with aquatic macrophytes.

The planktonic *Eubosmina* spp. peaked during the BA. *Eubosmina*-dominated lakes are typically deeper than chydorid-dominated lakes. Although there is no depth correlation between *Eubosmina* and *Daphnia*, the latter can exhibit a preference for nutrient rich lakes with a higher specific conductivity. *Chydorus* spp. remain abundant, as the taxa can adapt to the gradual increase in primary production and decrease in ice cover that favors a more open water habitat, along with conductivity or nutrient changes. It should be noted that the abundance of *Daphnia* may be underrepresented, as the taxa is generally more poorly preserved than Bosminidae or Chydoridae exoskeletal sedimentary remains, with only their post-abdomens and ephippia identifiable. There is a reduction on the abundance of all *Alona* species apart from the eurybiotic *A. setulosa*, which like *A. circumfibriata*, can tolerate high conductivity.

During the YD, no cladoceran remains were evident in the sediment. Terrestrial and aquatic invertebrate and Coleopteran remains, mites and fibrous root mass indicated that the lake could have intermittently dried up. The onset of the early Holocene brought a wetter and warmer climate with the formation of a deeper lake indicated by a sharp increase in pelagic *Eubosmina* (>68%), and a gradual return of pioneering benthic and littoral species, such as *Chydorus*, *Alona circumfimbriata* and *Alona intermedia* that are tolerant of changes and increases in conductivity. The decline of *Eubosmina* around 7000 cal yr BP indicates a marked decrease in lake depth and the onset of a drier and cooler climate (2). This is supported by the increase in benthic and littoral *Alona* species, many of which are still present in the lake today.

**Chironomids:**

**Methods:** Volumetric samples of lake sediment were processed for subfossil chironomid head capsules following standard procedures for non-calcareous sediments (19). First, sediments were deflocculated by gently warming in a 5% KOH solution for ~30 min. Sediments were then rinsed with distilled water on a 95-μm mesh sieve. The remaining material retained on the sieve was then transferred into a beaker with ~25 mL of distilled water. Successive aliquots were next poured into a Bogorov counting tray and all head capsules were handpicked using fine forceps at ~25X magnification. To ensure all subfossil chironomids were collected, multiple passes (i.e. two focused on the surface and two focused on the bottom of the Bogorov tray) were made through each tray. Subfossils were then placed on a cover glass and permanently mounted on a slide using Entellan®. Chironomids were identified with brightfield illumination and a compound microscope at either 200X or 400X magnification. Identifications of head capsules followed standard subfossil taxonomic keys for chironomid subfamilies (20). Coarser taxonomic groupings defined by the transfer function applied (21) were typically followed. For example, *Cricotopus* sp. and *Orthocladius* sp. were combined and most members of Tanytarsini were recognized as Tanytarsina (undifferentiated) (21). Distinctions of taxa such as *Parakiefferiella*-sp. B, *Zalutschia* sp., and Tanypodinae also were based on the taxonomy of the northwest North American training set and previous eastern Beringia chironomid reconstructions (22-24).

A minimum count of 50 individual chironomids per sediment interval was targeted for environmental inferences through time (25-27). Five of 51 samples analyzed, all within the Holocene, did not reach the targeted minimum count, but are included in the reconstruction given that each exceeded 30 individual chironomids (n = 32, 36, 37, 41, and 48 individuals). All identifiable chironomids were included as a percentage of the total count and past temperatures were estimated from the chironomid assemblages using the northern North American inference model (21). In order to evaluate whether the chironomid assemblages for the St. Paul Island sediment core had appropriate analogues for paleo climate reconstruction, each fossil interval was passively positioned on a Principal Components Analysis (PCA) of the Fortin et al. (2015) training set (SI 7). A robust reconstruction is reflected by the chironomid assemblages transitioning along the modern-day temperature gradient (28) reflected by the calibration-set (21). Goodness-of-fit for the chironomid-based paleo climate reconstruction was evaluated by a Canonical Correspondence Analysis (CCA) singly constrained to mean-July air temperature. Fossil intervals with a squared residual distance value within the most extreme 5 % of values in the Fortin et al. (2015) model were considered to have a poor fit with respect to temperature, those within the most extreme 15 % a ‘fair’ fit, and those beyond 15 % were considered non-analogue (SI 7). Taxon relative abundances were chi-square transformed. All analyses were completed in R version 3.2.2 (29) using the rioja, analogue, and vegan libraries.

**Results:** Subfossil chironomid assemblages at Lake Hill demonstrate substantial compositional changes, often abrupt and of a large magnitude, between ~17,000 cal yr BP and the early Holocene (SI 6). Temperature inferences during HS1 are generally the lowest of the Lake Hill record and average ~ 5 °C. Overall, a trend toward slightly warmer temperatures is observed throughout HS1 and coincides with increasing abundances of Tanytarsini. The dominant chironomid taxa during HS1, such as *Hydrobaenus*/*Oliveridia*, *Micropsectra*-type, and *Abiskomyia*, are more cold-adapted taxa typically observed in oligotrophic lakes from higher latitudes (21, 23). *Abiskomyia* was also common and abundant during the LGM at Burial Lake in western Alaska (22). Similarly, *Hydrobaenus*/*Oliveridia* often exceeded 40% abundance between ~17,000 and 15,500 cal yr BP at Hanging Lake in easternmost Beringia (24). Only four occurrences of Chironomini taxa (i.e. *Stictochironomus*, *Chironomus*, and *Sergentia*), generally observed during warmer conditions (30), were observed during HS1. This, along with the abundance of cold stenotherms, supports the extreme nature of HS1 climate compared to later periods at Lake Hill.

The BA period at Lake Hill represents an abrupt ~1000-year transition from HS1 to the start of the YD at ~14,400 cal yr BP. On average, temperature inferences during the BA are ~2-3 °C higher than inferred temperatures of the HS1 or YD. A consistent warming trend from ~5 °C to ~8 °C, comparable to that of the YD-early Holocene transition at Lake Hill, is inferred from the chironomid assemblages. Overall, BA chironomid assemblages are more diverse than those during HS1. While cold-adapted taxa, such as *Micropsectra*-type, *Protanypus*, and *Paracladius,* still occur during the BA, several taxa appear in the Lake Hill record for the first time, including *Zalutschia*-type A, *Procladius*, and *Corynoneura oliveri*-type, and *Cryptochironomus*. These taxa are generally recognized as having intermediate temperature optima in the training set (21). *Procladius* is also as a predatory chironomid, likely from a taxonomically-broad group, and exists in a wide range of limnological conditions across lakes in northern North America where mean July air temperatures typically exceed 8 °C (21). *Psectrocladius* (*Psectrocladius*), a taxon typical of warmer sites of shallow to moderate depth in northwestern North American, also becomes common at Lake Hill during the BA and suggests more moderate environmental conditions during the BA compared to HS1. *Psectrocladius* (*Psectrocladius*) also appeared for the first time in the Burial Lake record at ~14,000 cal yr BP (22).

Chironomid assemblages during the YD show large shifts in dominant taxa (i.e. *Paracladius*, *Sergentia*, *Zalutschia*-type A); although, with only four chironomid samples from the YD at Lake Hill it is a challenge to interpret environmental conditions during this period. Chironomid-inferred temperatures suggest that the start and end of the YD was slightly cooler than the BA, by only ~1 °C. However, that value is well within the typical sample-specific error (±1.5 °C) of temperature inferences from the model used (21). A greater sample resolution is needed during the YD to better recognize chironomid assemblage patterns. However, numerous samples were examined from this section with only trace amounts of diatoms found.

The early Holocene between ~11,700 and 7,000 cal yr BP at Lake Hill is defined by a marked transition in chironomid assemblages compared to pre-Holocene periods. Generally, more cold-adapted taxa that were abundant earlier are no longer observed in the Lake Hill core. Average chironomid-inferred temperatures during the early Holocene are ~9 °C and stable. Chironomini taxa, such as *Stictochironomus* and *Chironomus,* are common throughout. *Chironomus* reaches ~65% abundance prior to 7,000 cal yr BP. Additionally, *Stictochironomus*, *Psectrocladius* (*Psectrocladius*) and Tanytarsini are also common and relatively abundant during the early Holocene. These taxa all tend to show broader environmental and climatic tolerances (23) and suggest that the early Holocene at Lake Hill may have persisted for longer than previously recognized in eastern Beringia (22, 24). After ~7,000 cal yr BP, *Chironomus* abruptly is extirpated from the sediment record following its peak abundance of ~65% and as stable Holocene conditions develop at Lake Hill *Chironomus* likely lost its competitive advantage as an early colonizer. Between 7000 cal yr BP and modern times, temperature inferences are stable at around ~10 °C. This is the result of assemblages being composed of ~80-95% Tanytarsini, a taxon with a broad taxonomic designation in the training set from northern North America (21). This limits its usefulness in environmental reconstructions when members of Tanytarsini dominate. It should be noted that most Tanytarsini at Lake Hill during the Holocene belong to the genus *Paratanytarsus*, specifically *P. pencillatus*-type (not shown). This taxon is often observed during warmer conditions and may be associated with macrophytes (20, 31).

**Isotopic and elemental analyses:**

**Methods:** The oxygen isotope compositions (expressed as δ^18^O values) of chironomid head capsules were processed and analyzed at the Alaska Stable Isotope Facility (ASIF), University of Alaska Fairbanks using previously published protocols (32). Samples were processed from core sections at a ~4 cm resolution. After freeze-drying, samples were placed in an auto-sampler attached to a TCEA-IRMS system for analysis. All δ^18^O values are expressed relative to Vienna Standard Mean Ocean Water (VSMOW) and have an analytical precision of 0.1 per mil (‰), consistent with previously published protocols (4).

Bulk sediment samples were taken from the core at 16 cm intervals for elemental analyses. Methods followed previously published protocols (6, 33) for analyses total organic carbon (TOC) and total nitrogen (TN), and calculation of C:N ratios using a Costech ESC 4010 elemental analyzer interfaced via a ThermoConflo III to a Thermo DeltaV IRMS. Analytical precisions for TOC, and TN values were assessed using previously published protocols (6, 33) and were 2%, and 1% respectively.

**Results:** The δ^18^O values of chironomids strongly reflect the δ^18^O values of the water they live in (32, 34) and can be used to reconstruct δ^18^O values of past lake waters and climate (2, 33). Based on the findings from the modern lake water isotopes (above) our interpretation of the complete δ^18^O record from Lake Hill (Fig. 2h), which is consistent with previously published findings (2), is that higher δ^18^O values in the record are primarily driven by increased evaporative losses and lake level declines. Conversely, relatively low δ^18^O values at Lake Hill reflect decreased evaporative losses from the lake. From ~17,000 years ago to the end of HS1 the δ^18^O values generally increase, reflecting greater evaporative influences on the lakes hydrology, which is consistent with some of the other proxies indicating a decrease in lake levels over this time period (i.e. the decrease in planktonic diatoms and the eventual presence of the cladoceran species (*A. circumfimbriata*) that is more tolerant of higher lake water conductivity and salinity levels (35) that can occur due to evaporative losses from a lake. At ~14,500 years ago, leading into the BA, the δ^18^O values in Lake Hill decrease indicating a decrease in evaporative loses from the lake and taken along with the other hydrologic proxy date during the BA indicates that lake levels increased during this period. Towards the end of the BA δ^18^O values increase going into the YD, indicating a resumed lake level decrease through an increased evaporative loss of lake water. The δ^18^O values in Lake Hill are relatively low between ~12,000 and ~8,000 years ago but increase after this time, which has previously been interpreted as indicating increased evaporative losses from the lake following the early Holocene (2).

**Archeological and paleontological data:**

The archaeological data used in this paper, particularly Fig. 2b, were compiled from previously published records (36, 37). They comprise all securely dated archaeological components in Eastern Beringia (deglaciated parts of Alaska, Yukon Territory and adjacent areas) (n= 75). These dates were calibrated using Intcal13 (38), and a cumulative probability density graph was created in OxCal to serve as a proxy for human occupation density from the earliest securely dated site (Swan Point, at ~14,200 cal yr BP) to 10,000 cal yr BP. It has been shown that taphonomic biases, among other biases, affect using radiocarbon dated sites as proxies for population (39, 40), but our sample window (14,200-10,000 cal yr BP) is only minimally affected by the empirical taphonomic model expectations, which increase after ~5000 cal yr BP. These sites have been intensively sampled for this time period, particularly the interior Alaskan components (n=42). Calibration effects (plateaus and steps) can also influence summed probability distribution plots (40). Only one such plateau affects this time range, 12,400-10,700 cal yr BP, well after the initial occupation of Eastern Beringia. With these caveats, Fig. 2 archaeological component density is for heuristic purposes, to compare broad patterns of human colonization and density changes through the late Pleistocene and earliest Holocene.

The moose and horse data for interior Alaska were compiled from previously published records. These dates were all calibrated using Intcal09 (38). These calibrated dates were then assigned to 500-year bins to estimate a frequency distribution for both taxa (Figure 2).

**SI References:**

1. Wang Y*, et al.* (In Press) The southern coastal Beringian land bridge: cryptic refugium or pseudo refugium for woody plants during the Last Glacial Maximum? *Journal of Biogeography*.

2. Graham RW*, et al.* (2016) Timing and causes of mid-Holocene mammoth extinction on St. Paul Island, Alaska. *Proceedings of the National Academy of Sciences of the United States of America* 113(33):9310-9314.

3. Portal IHDaD (2017) <http://catalog.northslope.org/catalog/entries/4875-imiq-hydroclimate-database-and-data-portal>.

4. Booth AL, Wooller MJ, Howe T, & Haubenstock N (2010) Tracing geographic and temporal trafficking patterns for marijuana in Alaska using stable isotopes (C, N, O and H). *Forensic Science International* 202(1-3):45-53.

5. G. B (2017) <http://www.waterisotopes.org>.

6. Wooller MJ*, et al.* (2012) An ~11,200 year paleolimnological perspective for emerging archaeological findings at Quartz Lake, Alaska. *Journal of Paleolimnology* 48(1):83-99.

7. Battarbee RW (2001) Diatoms, Tracking environmental change using lake sediments. *Terrestrial, Algal and Siliceous Indicators*, eds Smol JP, Birks HJB, & WM L (Kluwer Academic Publishers), Vol 3, pp 155-202.

8. Antoniades D, Hamilton PB, Douglas MSV, & Smol JP (2008) Diatoms of North America: The freshwater floras of Prince Patrick, Ellef Ringnes, and Northern Ellesmere Islands from the Canadian Arctic Archipelago. *Iconographica Diatomologica* 17:1-649.

9. Cumming BF, Wilson SE, Hall RI, & Smol JP (1995) *Diatoms from British Columbia (Canada) lakes and their relationship to salinity, nutrients and other limnological variables* (Cramer).

10. Fallu M, Allaire N, & Pienitz R (2000) *Freshwater diatoms from northern Québec and Labrador (Canada): species–environment relationships in lakes of boreal forest, forest– tundra and tundra regions* (Cramer).

11. Gregory-Eaves I., Smol J.P., Finney B.P., & M.E. E (1999) Diatom-based transfer functions for inferring past climatic and environmental changes in Alaska, U.S.A. . *Arctic, Antarctic and Alpine Research* 31(4):353-365.

12. Saros JE*, et al.* (2012) Climate-induced changes in lake ecosystem structure inferred from coupled neo- and paleoecological approaches. *Ecology* 93(10):2155-2164.

13. Korhola A & Rautio M (2001) Cladocera and other branchiopod crustaceans. Tracking environmental change using lake sediments. *Zoological Indicators*, eds Smol JP, Birks HJB, & Last WM (Kluwer Academic Publishers), Vol 4, pp 5-41.

14. Alonso M (1996) *Crustacea Branchiopoda. Fauna Iberica* (Museo Nacional de Ciencias Naturales).

15. Flӧssner D (1972) *Krebstiere, Crustacea: Kiemen- und Blattfüsser, Branchiopoda, Fischlduse, Branchiura. Die Tierwelt Deutschlands* (Fisher) p 501.

16. Frey DG (1958) The late-glacial cladoceran fauna of a small lake. *Arch. Hydrobiol* 54:209- 275.

17. Smirnov NN (1971) *Chydoridae of the world fauna. Fauna USSR. Crustacea 1 No. 2* (Leningrad) p 529.

18. Nykänen M, Vakkilainen K, Liukkonen M, & Kairesalo T (2009) Cladoceran remains in lake sediments: A comparison between plankton counts and sediment records. *Journal of Paleolimnology* 42(4):551-570.

19. Walker IR (2001) Midges: Chironomidae and related Diptera. . *Tracking Environmental Change Using Lake Sediments. Zoological Indicators*, eds Smol JP, Birks HJB, & Last WM (Kluwer Academic Publishers), Vol 4, pp 43–66.

20. Brooks SJ, Langdon PG, & Heiri O (2007) *The identification and use of palaearctic chironomidae larvae in palaeoecology* (Quaternary Research Association, London) p 276.

21. Fortin MC*, et al.* (2015) Chironomid-environment relations in northern North America. *Journal of paleolimnology* 54(2-3):223-237.

22. Kurek J, Cwynar LC, Ager TA, Abbott MB, & Edwards ME (2009) Late Quaternary paleoclimate of western Alaska inferred from fossil chironomids and its relation to vegetation histories. *Quaternary Science Reviews* 28(9-10):799-811.

23. Barley EM*, et al.* (2006) A northwest North America training set: distribution of freshwater midges in relation to air temperature and lake depth. *Journal of Paleolimnology* 36:295–314.

24. Kurek J, Cwynar LC, & Vermaire JC (2009) A late Quaternary paleotemperature record from Hanging Lake, northern Yukon Territory, eastern Beringia. *Quaterrnary Research* 72:246–257.

25. Heiri O & Lotter AF (2001) Effect of low count sums on quantitative environmental reconstructions: an example using subfossil chironomids. *Journal of Paleolimnology* 26:343–350.

26. Larocque I (2001) How many chironomid head capsules are enough? A statistical approach to determine sample size for palaeoclimatic reconstructions. *Palaeogeography, Palaeoclimatology and Palaeoecology* 172:133–142.

27. Quinlan R & Smol JP (2001) Setting minimum head capsule abundance and taxa deletion criteria in chironomid-based inference models. *Journal of Paleolimnology* 26:327–342.

28. Medeiros AS, Gajewski K, Porinchu DF, Vermaire JC, & Wolfe BB (2015) Detecting the influence of secondary environmental gradients on chironomid-inferred paleotemperature reconstructions in northern North America. *Quaternary Science Reviews* 124:265-274.

29. Team RC (2015) R: A language and environment for statistical computing. R Foundation for Statistical Computing.

30. Medeiros AS & Quinlan R (2011) The distribution of the Chironomidae (Insecta: Diptera) along multiple environmental gradients in lakes and ponds of the eastern Canadian Arctic. *Canadian journal of fisheries and aquatic sciences* 68(9):1511-1527.

31. Langdon PG, Ruiz Z, Wynne S, Sayer CD, & Davidson TA (2010) Ecological influences on larval chironomid communities in shallow lakes: implications for palaeolimnological interpretations. *Freshwater Biology* 55:531-545.

32. Wang Y, Francis DR, O'Brien DM, & Wooller MJ (2008) A protocol for preparing subfossil chironomid head capsules (Diptera: Chironomidae) for stable isotope analysis in paleoclimate reconstruction and considerations of contamination sources. *Journal of Paleolimnology* 40(3):771-781.

33. Wooller MJ*, et al.* (2012) Reconstruction of past methane availability in an Arctic Alaska wetland indicates climate influenced methane release during the past ~12,000 years. *Journal of Paleolimnology* 48(1):27-42.

34. Wooller MJ*, et al.* (2004) Quantitative paleotemperature estimates from δ18O of chironomid head capsules preserved in arctic lake sediments. *Journal of Paleolimnology* 31(3):267-274.

35. Thienpont JR*, et al.* (2013) Exploratory hydrocarbon drilling impacts to arctic lake ecosystems. *PLoS ONE* 8(11).

36. Potter B (2008) Radiocarbon Chronology of Central Alaska: Technological Continuity and Economic Change. *Radiocarbon* 50(2):181-204.

37. Potter BA, Holmes CE, & Yesner DR (2013) Technology and economy among the earliest prehistoric foragers in interior Eastern Beringia. *Paleoamerican Odyssey*, (Texas A&M Press), pp 81-103.

38. Reimer P*, et al.* (2009) IntCal09 and Marine09 radiocarbon age calibration curves, 0–50,000 years cal BP. *Radiocarbon* 51(4):1111–1150.

39. Weninger B, Edinborough K, Clare L, & Joris O (2011) Concepts of probability in radiocarbon analysi. *Documenta Praehistoricas* 38(1):1-20.

40. Williams A (2012) The use of summed radiocarbon probability distributions in archaeology: a review of methods. *Journal of Archaeological Science* 39:578-589.

SI 2: Top - St. Paul Island showing lakes where water samples were taken for stable oxygen and hydrogen isotope analyses. Bottom - Stable oxygen and hydrogen isotope data from lakes on St. Paul Island, Alaska (navy symbols), including Lake Hill (light green symbols). These data are shown alongside measured values of ground water (brown symbols) and winter precipitation (white symbols) along with modeled monthly values (pale blue symbols) and the modeled mean annual values (red circle). The line represents the Global Meteoric Water Line.


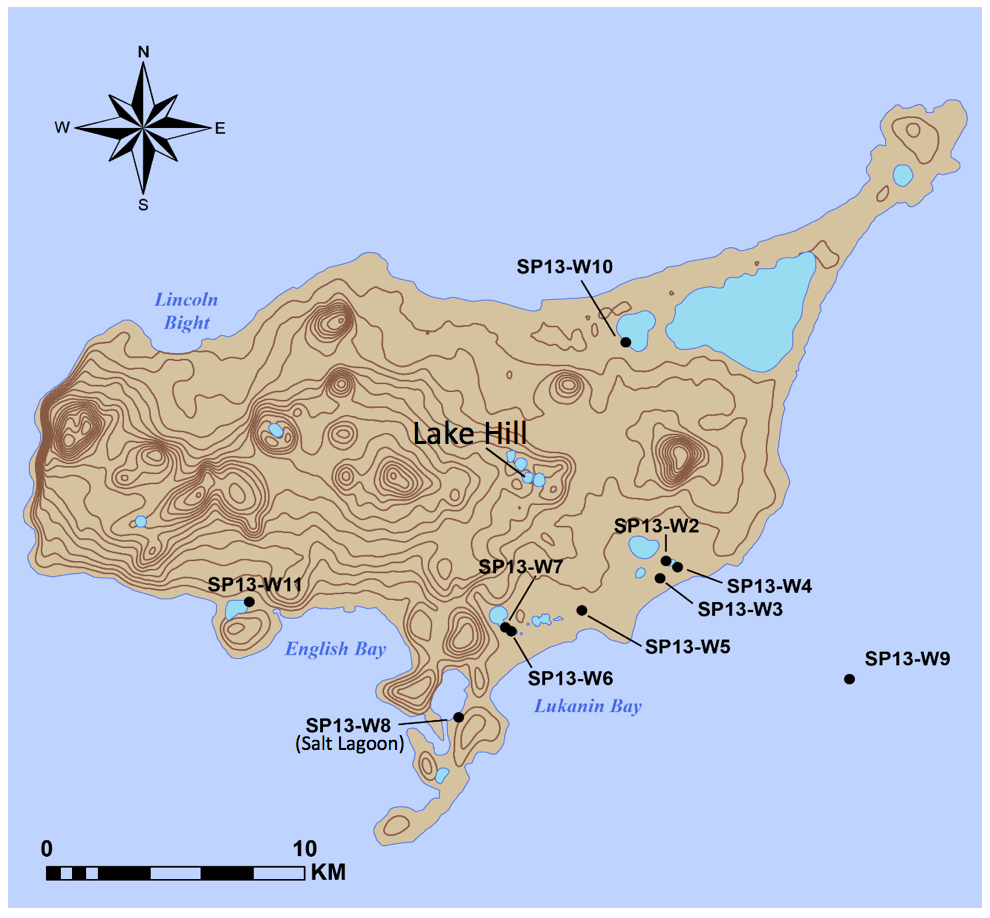

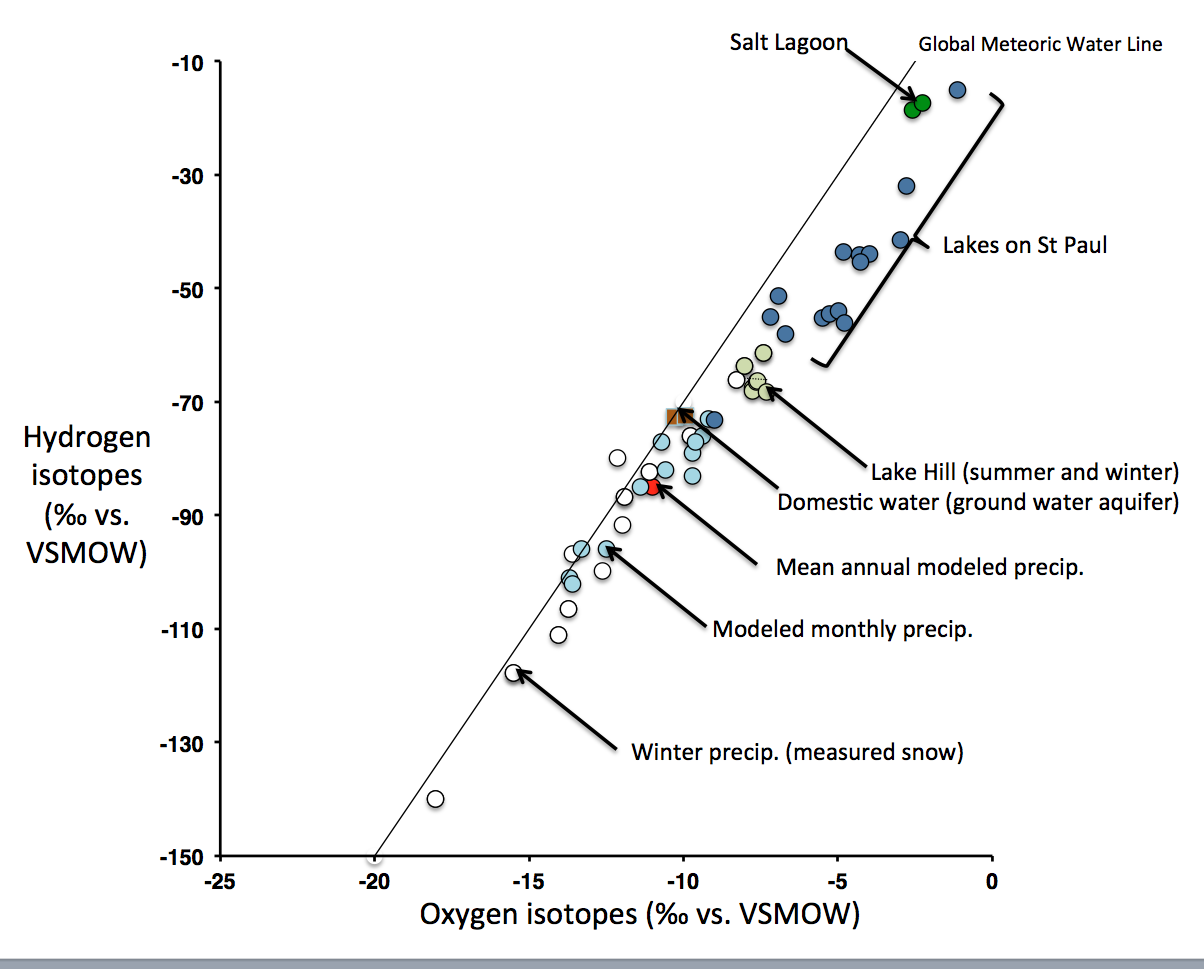


|  |
| --- |

SI 3: Table 1. YSI data collected March 2013 from an ice-covered Lake Hill, St. Paul Island.

| Elevation  (m a.s.l.) | Lake depth  (cm) | Lake water Temperature  (^o^C) | Salinity  (ppt) | pH | Ice thickness (cm) |
| --- | --- | --- | --- | --- | --- |
| 39 | 130 | 2.3 (+-1) | 0.07 (+-0.01) | 5.5 (+-0.3) | 75 |

SI 4: Marine diatom remains from section 869-981 cm of the Lake Hill core: A-C) illustration of the extent of the degradation of the frustules; D-E) *Paralia sulcata*; F) *Thalassionema nitzschioides*; G-H) *Cosmiodiscus insignis*; I) *Coscinodiscus marginatus*. Table 2. List of marine species identified in section 869-981 cm of the St-Paul sediment core. Xs mark samples in which each taxon was found.


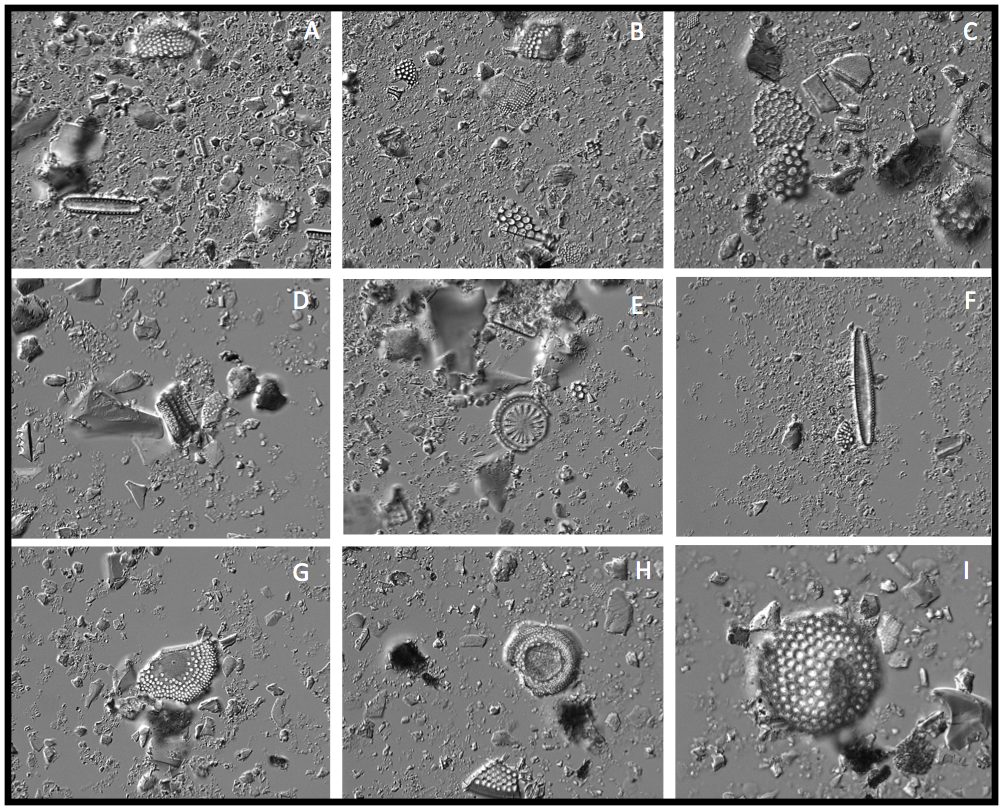


| **Species names** | 869 cm | 885 cm | 901 cm | 917 cm | 933  cm | 949 cm | 965 cm | 981 cm |
| --- | --- | --- | --- | --- | --- | --- | --- | --- |
| *Actinoptychus senarius* (Ehrenberg) Ehrenberg, 1843 | x |  | x | x | x | x | x |  |
| *Actinicyclus sp*. (as in Gladenkov 2003* Pl.1, Fig.12) |  |  |  |  |  | x |  |  |
| *Coscinodiscus marginatus* Ehrenberg, 1841 | x | x | x | x | x | x | x |  |
| *Coscinodiscus pustulatus* Mann, 1907 | x |  |  | x | x | x |  | x |
| *Cosmiodiscus insignis* Jousé, 1961 | x | x | x | x | x | x | x | x |
| *Cymatosira debyi* Tempère & Brun in Brun & Tempère, 1889 |  |  |  | x |  |  |  |  |
| *Delphineis angustata* (Pantocsek) Andrews 1977 |  | x |  |  | x |  | x |  |
| *Delphineis simonsenii* (Mertz) Akiba, 1985 |  |  |  |  | x |  |  |  |
| *Dictyocha fibula* Ehrenberg, 1839 |  |  |  | x | x |  |  |  |
| *Hyalopyxis concava* (Sheshukova) Makarova, 1989 | x | x | x | x | x | x |  | x |
| *Neodenticula kamtschatica* (M.M.Zabelina) F.Akiba & Y.Yanagisawa 1986 | x | x | x | x | x | x | x | x |
| *Paralia sulcata* (Ehrenberg) Cleve 1873 | x | x | x | x | x | x |  | x |
| *Porosira glacialis* (Grunow) Jørgensen 1905 | x |  |  |  |  |  |  |  |
| *Rhaphoneis amphiceros* (Ehrenberg) Ehrenberg 1844 |  |  |  |  |  | x |  | x |
| *Thalassionema nitzschioides* (Grunow) Mereschkowsky 1902 | x | x | x | x | x | x | x | x |
| *Thalassiosira antiqua* (Grunow) Cleve-Euler |  |  |  | x |  | x |  |  |
| *Thalassiosira dolomatovae* Oreshkina *in* Gladenkov 1992 | x | x |  |  |  |  |  |  |
| *Thalassiosira gravida* Cleve 1896 | x | x | x |  | x | x |  | x |
| *Thalassiosira latimarginata* Makarova, 1975 |  | x | x |  | x |  |  |  |
| *Thalassiosira nativa* Sheshukova-Poretskaya, 1964 | x |  | x | x | x | x | x | x |
| *Thalassiosira marujanica* Sheshukova-Poretzkaya sensu Akiba, 1986 |  |  |  |  |  | x |  |  |

*Gladenkov, A.Y. 2003. Diatom Biostratigraphy of the Neogene Milky River Formation, Alaska Peninsula, Southwestern Alaska. *Proceedings of the California Academy of Sciences* 54(3): 27-64.

SI 5: C:N ratio of sediment from Lake Hill core vs. calendar years before present.


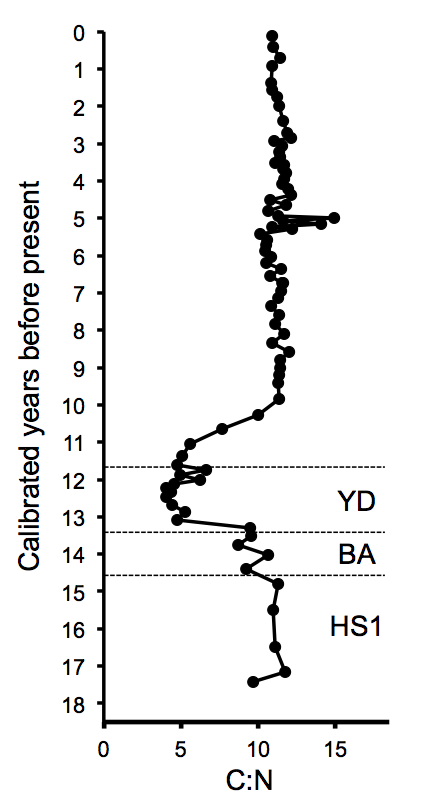


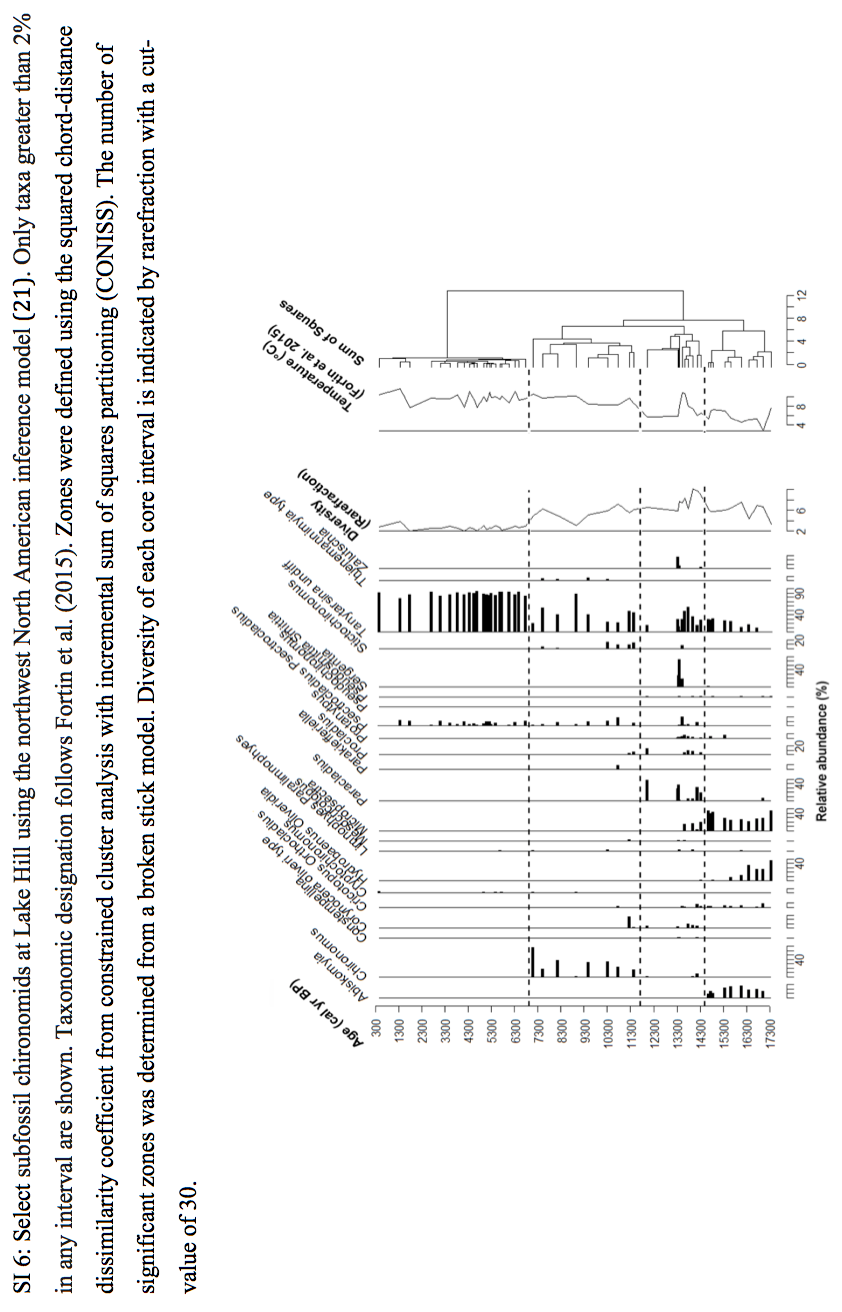


SI 7: Left - The chironomid assemblages of the St. Paul Island sediment core positioned passively (red line) ontop of a Principal Components Analysis (PCA) of a published (21) calibration-set. Isotherms of mean-July air temperature are indicated. Right - Goodness-of-fit of chironomid assemblages for each sediment interval compared to a previously published model (21). The squared residual length of subfossil samples is determined by a difference from analogues in the published (21) dataset under a Canonical Correspondence Analysis (CCA) constrained solely against July temperature. Samples that had a squared residual distance value within the most extreme 5 % of values in the Fortin et al. (2015) model were considered to have a poor fit, those within the most extreme 15 % a ‘fair’ fit, and those beyond the most extreme 15 % were considered non-analogues with respect to temperature.


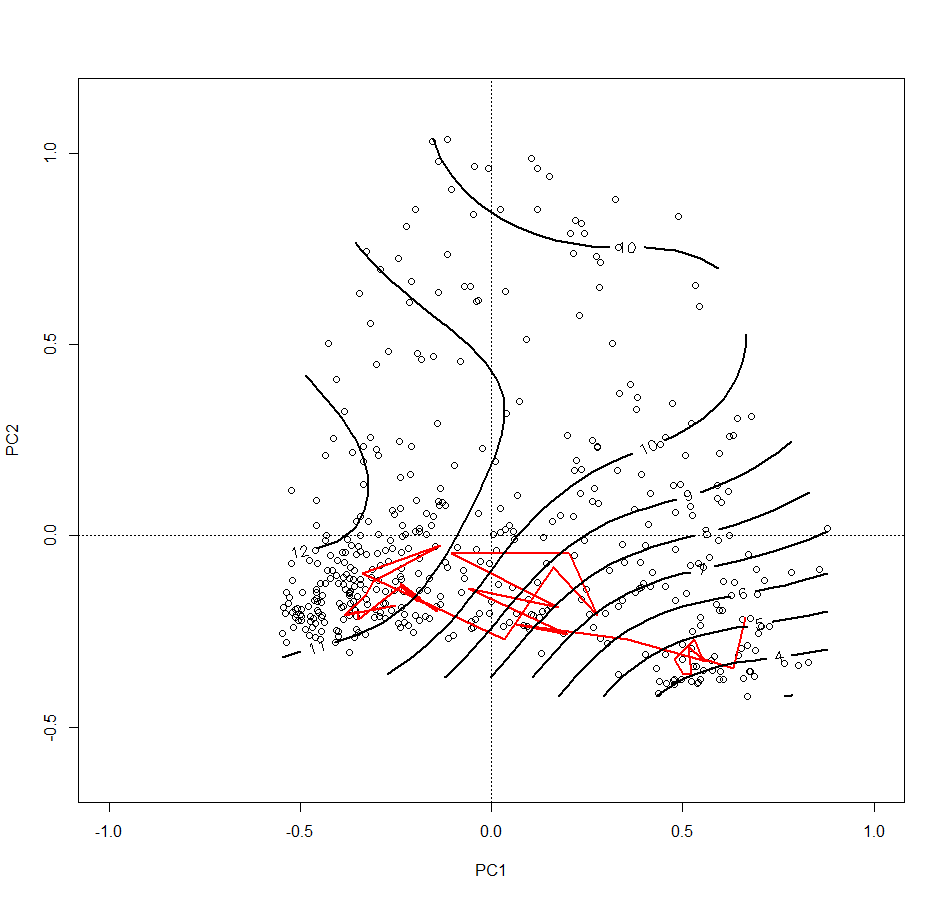


SI 8: Stratigraphic diagram of sedimentary diatom assemblages from the Lake Hill core plotted against time (ages in cal yr BP). Only the most abundant species are shown and are ordered according to the timing of their downcore occurrences. The percentage of the assemblages comprised of planktonic and tychoplanktonic taxa is also shown (left). The grey bar represents the portion of the core where marine diatom fragments were identified (see also SI 4).


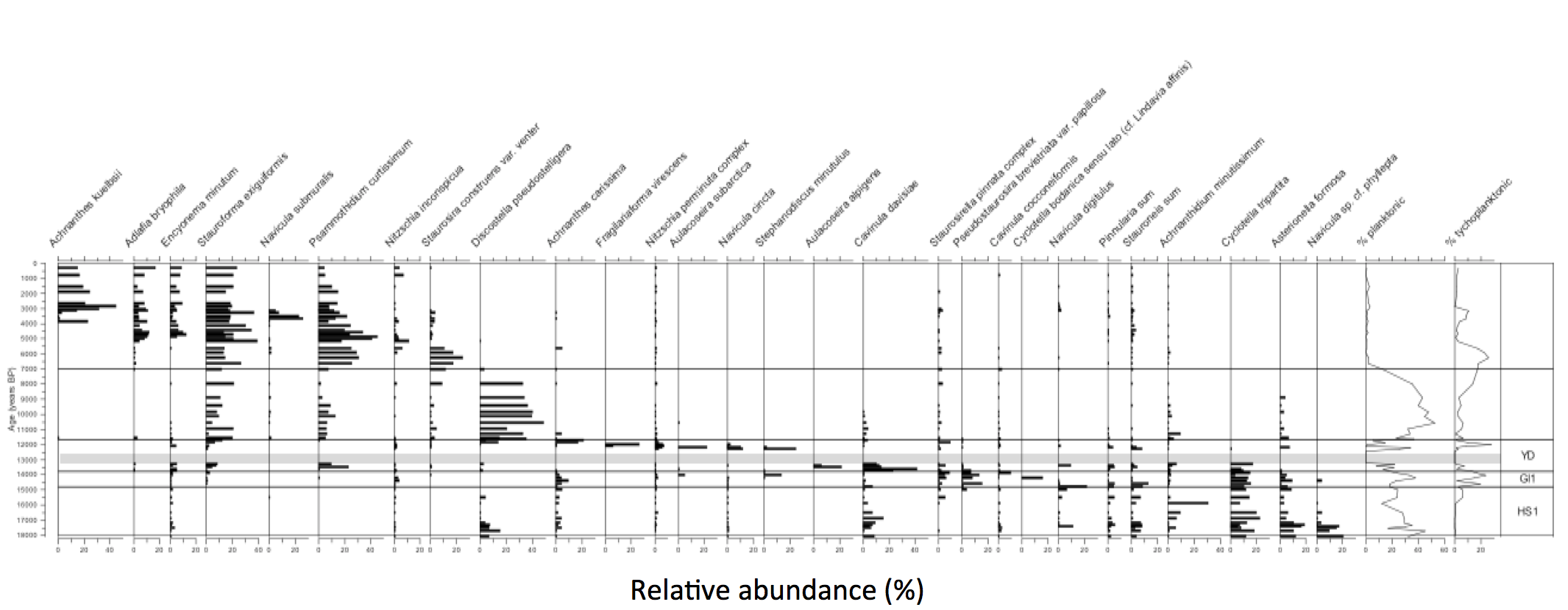


SI 9: Examples of fresh-water diatom taxa from Lake Hill core. 1-3 *Achnanthes kuelbsii*; 4-6 *Adlafia bryophila*; 7-9 *Encyonema minutum*; 10-12 *Stauroforma exiguiformis*; 13-15 *Navicula submuralis*; 16-18 *Psammothidium curtissimum*; 19-22 *Staurosira construens* var. *venter*; 23-25 *Nitzschia inconspicua*; 26-29 *Discostella pseudostelligera*; 30-32 *Fragilariaforma virescens* 33-35 *Nitzschia perminuta*; 36-38 *Achnanthes carissima*; 39-43 *Aulacoseira subarctica*; 44-46 *Navicula cincta*; 47-49 *Stephanodiscus minutulus* 50-53 *Aulacoseira alpigena* 54-56 *Cavinula davisiae*; 57-61 *Staurosirella pinnata*; 62-66 *Pseudostaurosira brevistriata* var. *papillosa*; 67-69 *Cavinula cocconeiformis*; 70-72 *Navicula digitulus*; 73-76 *Cyclotella bodanica sensu lato* (cf. *Lindavia affinis*); 77-81 *Achnanthidium minutissimum*; 82-85 *Cyclotella tripartita*; 86-88 *Navicula* sp. cf. *phyllepta*; 89-91 *Asterionella formosa.*


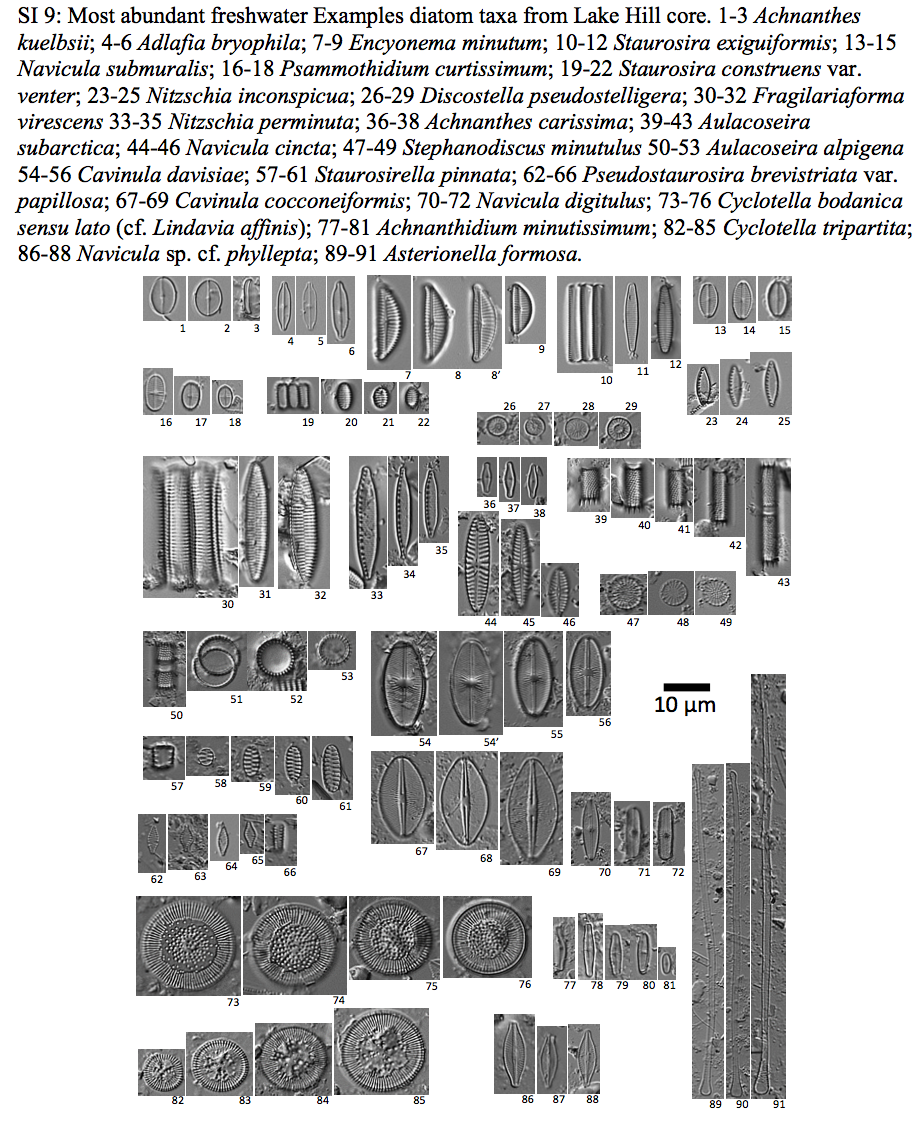


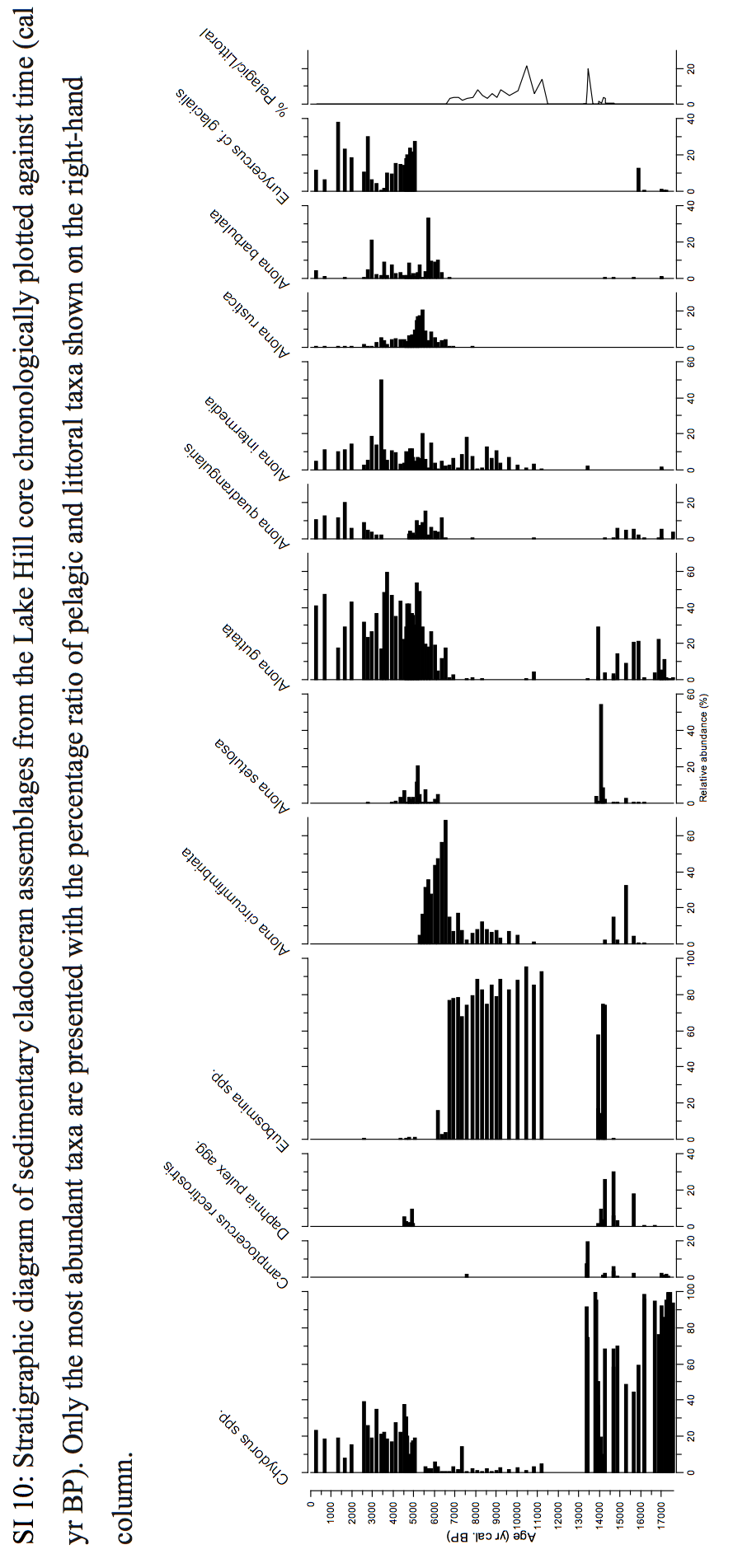


SI 11: Table 3. Published radiocarbon data for moose and horse from interior Alaska.

| **Site Code/Museum no** | **14C Date** | **1 Sigma** | **material** | **Published** |  |
| --- | --- | --- | --- | --- | --- |
| AM 2167 | 158 | 48 | *Alces* | Guthrie 2006 |  |
| AM 34670B | 262 | 44 | *Alces* | Guthrie 2006 |  |
| AM 5745 | 1158 | 52 | *Alces* | Guthrie 2006 |  |
|  | 1190 | 40 | *Alces* | Guthrie 2006 |  |
| AM 5703 | 1200 | 62 | *Alces* | Guthrie 2006 |  |
|  | 1250 | 30 | *Alces* | Guthrie 2006 |  |
| AM 2311 | 3401 | 79 | *Alces* | Guthrie 2006 |  |
| AM 8876 | 3539 | 58 | *Alces* | Guthrie 2006 |  |
|  | 3916 | 29 | *Alces* | Guthrie 2006 |  |
| AM 2037-A | 3941 | 52 | *Alces* | Guthrie 2006 |  |
| AM 2453 | 3983 | 65 | *Alces* | Guthrie 2006 |  |
| AM 1372 | 3992 | 54 | *Alces* | Guthrie 2006 |  |
| AM 34586 | 4164 | 56 | *Alces* | Guthrie 2006 |  |
|  | 4254 | 31 | *Alces* | Meiri et al. 2015 |  |
| AM 34594 | 4414 | 54 | *Alces* | Guthrie 2006 |  |
| AM 5019 | 4447 | 53 | *Alces* | Guthrie 2006 |  |
| AM 4732 | 5294 | 61 | *Alces* | Guthrie 2006 |  |
| AM 2161 | 5534 | 59 | *Alces* | Guthrie 2006 |  |
| AM 7527 | 5805 | 56 | *Alces* | Guthrie 2006 |  |
| AM 7527 | 5941 | 90 | *Alces* | Guthrie 2006 |  |
| AM 8743 | 6022 | 54 | *Alces* | Guthrie 2006 |  |
| AM 5279 | 6221 | 138 | *Alces* | Guthrie 2006 |  |
| AM 2164 | 6361 | 62 | *Alces* | Guthrie 2006 |  |
| AM 1426 | 6369 | 69 | *Alces* | Guthrie 2006 |  |
| AM 5280 | 6396 | 62 | *Alces* | Guthrie 2006 |  |
| AM 5023 | 6621 | 60 | *Alces* | Guthrie 2006 |  |
| AM 5283 | 6631 | 69 | *Alces* | Guthrie 2006 |  |
| AM 8651 | 6651 | 68 | *Alces* | Guthrie 2006 |  |
| AM 5269 | 6667 | 59 | *Alces* | Guthrie 2006 |  |
| AM 5311 | 6676 | 92 | *Alces* | Guthrie 2006 |  |
| AM 3905 | 6711 | 69 | *Alces* | Guthrie 2006 |  |
| AM 2161 | 6716 | 61 | *Alces* | Guthrie 2006 |  |
| AM 5270 | 6727 | 58 | *Alces* | Guthrie 2006 |  |
| AM 5308 | 6761 | 76 | *Alces* | Guthrie 2006 |  |
| AM 5309 | 6774 | 70 | *Alces* | Guthrie 2006 |  |
| AM 2310 | 6788 | 60 | *Alces* | Guthrie 2006 |  |
| AM 3886 | 6791 | 65 | *Alces* | Guthrie 2006 |  |
| AM 2650 | 6840 | 59 | *Alces* | Guthrie 2006 |  |
| AM 34602 | 6998 | 71 | *Alces* | Guthrie 2006 |  |
| AM 34587 | 7121 | 94 | *Alces* | Guthrie 2006 |  |
| AM 34602-B | 7128 | 104 | *Alces* | Guthrie 2006 |  |
| AM 1427B | 7162 | 73 | *Alces* | Guthrie 2006 |  |
| AM 9043 | 7484 | 53 | *Alces* | Guthrie 2006 |  |
| AM 33588 | 8839 | 83 | *Alces* | Guthrie 2006 |  |
| AM 5286 | 8856 | 79 | *Alces* | Guthrie 2006 |  |
| AM 34591 | 8858 | 80 | *Alces* | Guthrie 2006 |  |
| AM 34593 | 8877 | 77 | *Alces* | Guthrie 2006 |  |
| AM 34602-E | 9000 | 80 | *Alces* | Guthrie 2006 |  |
| AM 5284 | 9114 | 81 | *Alces* | Guthrie 2006 |  |
| AM 1361 | 9503 | 96 | *Alces* | Guthrie 2006 |  |
| AM 3459A | 9544 | 80 | *Alces* | Guthrie 2006 |  |
| AM 5735 | 9589 | 91 | *Alces* | Guthrie 2006 |  |
| AM 5365 | 9618 | 80 | *Alces* | Guthrie 2006 |  |
| AM 5312 | 9650 | 77 | *Alces* | Guthrie 2006 |  |
| AM 5702 | 9685 | 88 | *Alces* | Guthrie 2006 |  |
| AM 9080 | 9717 | 65 | *Alces* | Guthrie 2006 |  |
| AM 34589 | 9872 | 86 | *Alces* | Guthrie 2006 |  |
| AM 2651 | 9927 | 67 | *Alces* | Guthrie 2006 |  |
| AM 4323 | 10033 | 90 | *Alces* | Guthrie 2006 |  |
| AM 4034 | 10053 | 76 | *Alces* | Guthrie 2006 |  |
| AM 4023 | 10069 | 111 | *Alces* | Guthrie 2006 |  |
| AM 34592-D | 10103 | 92 | *Alces* | Guthrie 2006 |  |
| AM 5174/F:AM 144733 | 10756 | 64 | *Alces* | Guthrie 2006; Lanoe et al. in press |  |
|  | 10900 | 70 | *Alces* | Guthrie 2006 |  |
| AM 436776 | 11000 | 80 | *Alces* | Guthrie 2006 |  |
| AM 34590 | 11210 | 81 | *Alces* | Guthrie 2006 |  |
| AM 7779/F:AM 144730 | 11267 | 95 | *Alces* | Guthrie 2006; Lanoe et al. in press |  |
| AM 1387 | 11289 | 72 | *Alces* | Guthrie 2006 |  |
| AM 5172/F:AM 144735 | 11671 | 76 | *Alces* | Guthrie 2006; Lanoe et al. in press |  |
| AM 34592-C | 11678 | 81 | *Alces* | Guthrie 2006 |  |
| AM 5282/F:AM 144729 | 11790 | 117 | *Alces* | Guthrie 2006; Lanoe et al. in press |  |
| AM 1121/F:AM 144734 | 11947 | 89 | *Alces* | Guthrie 2006; Lanoe et al. in press |  |
| AM 3228 | 11988 | 82 | *Alces* | Guthrie 2006 |  |
| AM 2165 | 12022 | 153 | *Alces* | Guthrie 2006 |  |
| AM 5244/F:AM 144731 | 12201 | 85 | *Alces* | Guthrie 2006; Lanoe et al. in press |  |
| 15791 | 11950 | 100 | *Equus* | Krasinski and Haynes 2011; Lanoë and Holmes 2016 |  |
| AMNH F:AM 142429 | 12310 | 45 | *Equus* | Leonard et al. 2007; Fox-Dobbs et al. 2008; Rabanus et al. 2017 |  |
| F:AM-6735 | 12380 | 120 | *Equus* | Guthrie 2006 |  |
| A-46-34/F:AM 144736 | 12482 | 80 | *Equus* | Guthrie 2006; Lanoe et al. in press |  |
| A-144-9422/F:AM 144737 | 12510 | 130 | *Equus* | Guthrie 2006; Lanoe et al. in press |  |
| A-6159 | 12560 | 140 | *Equus* | Guthrie 2006 |  |
| AMNH F:AM 142423 | 12560 | 50 | *Equus* | Leonard et al. 2007; Fox-Dobbs et al. 2008; Rabanus et al. 2017 |  |
| A-144-9399/F:AM 144738 | 12580 | 140 | *Equus* | Guthrie 2006; Lanoe et al. in press |  |
| A-216-6846/F:AM 144739 | 12710 | 170 | *Equus* | Guthrie 2006; Lanoe et al. in press |  |
| F:AM-6282 | 12840 | 140 | *Equus* | Guthrie 2006 |  |
| A-4339/F:AM 144740 | 12860 | 140 | *Equus* | Guthrie 2006; Lanoe et al. in press |  |
| A-119no9 | 13250 | 160 | *Equus* | Guthrie 2006 |  |
| A-1005 | 13270 | 150 | *Equus* | Guthrie 2006 |  |
|  | 13350 | 120 | *Equus* | Sattler et al. 2001; Krasinski, 24, 28, 51, 103 |  |
|  | 13640 | 410 | *Equus* | Guthrie 2006, Krasinski, 25, 33, 51, 74, 84 |  |
| A-276/F:AM 144741 | 13940 | 160 | *Equus* | Guthrie 2006; Lanoe et al. in press |  |
| A-144-6987 | 14000 | 160 | *Equus* | Guthrie 2006 |  |
| A-237-10198 Goldstream | 14120 | 180 | *Equus* | Guthrie 2006 |  |
| A-558-2402/F:AM 144742 | 14260 | 160 | *Equus* | Guthrie 2006; Lanoe et al. in press |  |
| A-274 | 14300 | 160 | *Equus* | Guthrie 2006 |  |
| A-278-5640 | 14620 | 170 | *Equus* | Guthrie 2006 |  |
| F:AM 142421 | 14860 | 60 | *Equus* | Fox-Dobbs et al. 2008; Leonard et al. 2007 |  |
| A-160-6810 | 14990 | 190 | *Equus* | Guthrie 2006 |  |
| UA97-061-221 | 15150 | 70 | *Equus* | Potter 2005; Krasinski and Haynes 2011 |  |
